# Microbial extracellular vesicles from min pigs remodel macrophage polarization via STING to sustain intestinal immune homeostasis

**DOI:** 10.1080/19490976.2026.2620126

**Published:** 2026-01-27

**Authors:** Zhendong Sun, Zichuan An, Weichen Hong, Chenpeng He, Jiaxin Liu, Yupu Wang, Chenyu Xue, Na Dong

**Affiliations:** a The Laboratory of Molecular Nutrition and Immunity, College of Animal Science and Technology, Northeast Agricultural University, Harbin, People's Republic of China

**Keywords:** *Streptococcus hyointestinalis*, STING, macrophage polarization, extracellular vesicles, microbiota–host interactions, intestinal immune homeostasis

## Abstract

Intestinal immune homeostasis is crucial for intestinal function and health. Increasing evidence suggests that certain gut microbiota can enhance the host's intestinal immune regulatory capacity. However, the mechanisms by which the microbiota confers beneficial traits and robust immunity to the host, as well as the cross-species reproducibility of these effects, remain unclear. This study, through multi-omics integration comparison and functional validation, revealed that *Streptococcus hyointestinalis* from Min pigs regulates macrophage polarization homeostasis by targeting and inhibiting the excessive activation of the STING signaling pathway and its downstream pro-inflammatory cascade reactions through its extracellular vesicles (EVs), thereby shifting them toward the M2 phenotype. This process ensures the integrity of the intestinal barrier and alleviates colitis induced by the combined effects of low temperature and sodium sulfate-induced colitis (DSS). Notably, in *Sting*^*-/-*^ mice, the EV-mediated intestinal protective effect was eliminated, confirming its targeted efficacy. Our data reveal a microbial EV‒STING‒macrophage axis in which symbiotic bacterial exosomes promote reparative macrophage programs by regulating STING signaling and maintaining intestinal integrity under environmental stress. These findings reveal a novel host–microbiota communication pathway with therapeutic potential for the treatment of inflammation-driven intestinal diseases.

## GRAPHICAL ABSTRACT

**Figure:**
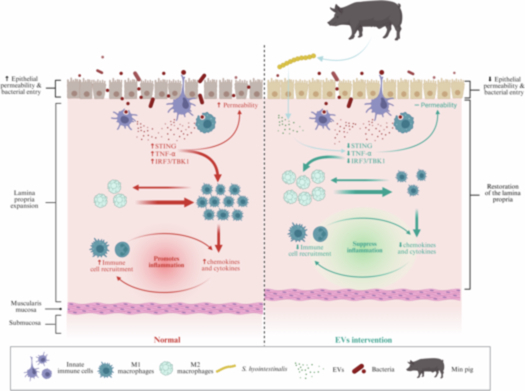

## Introduction

Gut immune homeostasis, which is critical for maintaining a healthy pathogen barrier and regulating inflammatory responses, is tightly controlled by immunoregulatory mechanisms established by microbes and their microbial products interacting with immune cells. Environmental stressors (e.g., low-temperature exposure) can disrupt this homeostasis, leading to increased susceptibility to intestinal inflammation. Previous studies have shown that cold exposure alters the gut microbial composition, compromises gut barrier integrity, and alters the balance of immune cell populations.^1^

Dysregulation of intestinal immune homeostasis underlies the pathogenesis of chronic inflammatory diseases such as inflammatory bowel disease (IBD), in which aberrant immune activation leads to tissue destruction and barrier dysfunction.^2-4^ Prolonged exposure of animals to cold environments can impair intestinal barrier integrity and alter the composition of the intestinal microbiota, but the mechanisms linking cold exposure, microbial regulation, and immune responses remain unclear. It is now well established that microbial products can modulate host immune responses, and bacterially secreted extracellular vesicles (EVs) have been recognized as key mediators of host‒microbe communication. The intestinal microbiota actively communicates with host immune cells through metabolites and EVs to fine-tune responses.^5^ Bacterial EVs rich in proteins, lipids, and nucleic acids have become effective immunomodulators that can affect a range of immune responses.^6^ Related studies have shown that EVs can participate in the occurrence and development of diseases by affecting the polarization state of macrophages and regulating the immune microenvironment.^7^ Especially in inflammatory diseases, EVs may be able to significantly attenuate the inflammatory response and protect intestinal tissues from damage caused by immune overreaction by modulating the activation of the cyclic guanosine monophosphate-adenylate synthase (cGAS)-interferon gene stimulating factor (STING) signaling pathway. *Lactobacillus* derived EVs reduce inflammation by inhibiting NF-κB activation, whereas pathogenic bacterial EVs exacerbate immune dysfunction.^8^ Despite these advances, the role of commensal bacteria-derived EVs in regulating STING-immune cell crosstalk under cold temperatures remains unexplored.

Macrophages are key mediators of innate immunity, dynamically switching between pro-inflammatory M1 and reparative M2 phenotypes, a process that is critical for resolving inflammation and repairing tissue damage. Dysregulated macrophage polarization is associated with inflammatory bowel disease (IBD), including colitis, in which excessive M1 activation exacerbates mucosal damage and inflammatory responses. The STING pathway, one of the key pathways known to generate responses to microbial pathogens and to regulate immune cell polarization, is overactivated under conditions of stress (e.g., hypoxia, extreme temperatures, or chemical insults), disrupting epithelial tight junctions, augmenting proinflammatory macrophage polarization, and perpetuating feedforward circuits of tissue injury.^9^^,^^10^ Recent studies have highlighted the STING pathway as one of the central regulators of macrophage polarization and the inflammation cascade. The activation of STING triggers the phosphorylation of TANK-binding kinase 1 (TBK1) and interferon-regulating factor 3 (IRF3), which in turn drives pro-inflammatory cytokine production and M1 polarization. Surprisingly, the STING pathway intersects with macrophage polarization: the activation of STING in macrophages amplifies IFN-β and IL-6 production and regulates the M1/M2 macrophage polarization balance.^11^^,^^12^ However, uncontrolled activation of this pathway leads to excessive inflammation and tissue damage.

In this study, the Min pig, a Chinese specialty cold-tolerant pig breed, was selected as a hypothermia-tolerant host phenotype to investigate the intrinsic mechanisms underlying its ability to maintain relatively stable intestinal immunity at low temperatures. Mechanistically, the cryotolerant host phenotype is supported by gut microbial-derived EVs that inhibit STING overactivation, thereby promoting M2 macrophage polarization and mucosal repair. Our study clarifies the microbial–EVs–STING–macrophage axis as a fundamental mechanism of immune homeostasis and advances the concept of microbial nanotherapies for the treatment of inflammatory diseases. By bridging microbial biology, innate immunity, and vesicle-mediated communication, this study redefines therapeutic strategies for IBD and related diseases.

## Results

### Min pigs still have strong intestinal immunity at low temperatures

**Figure 1.**
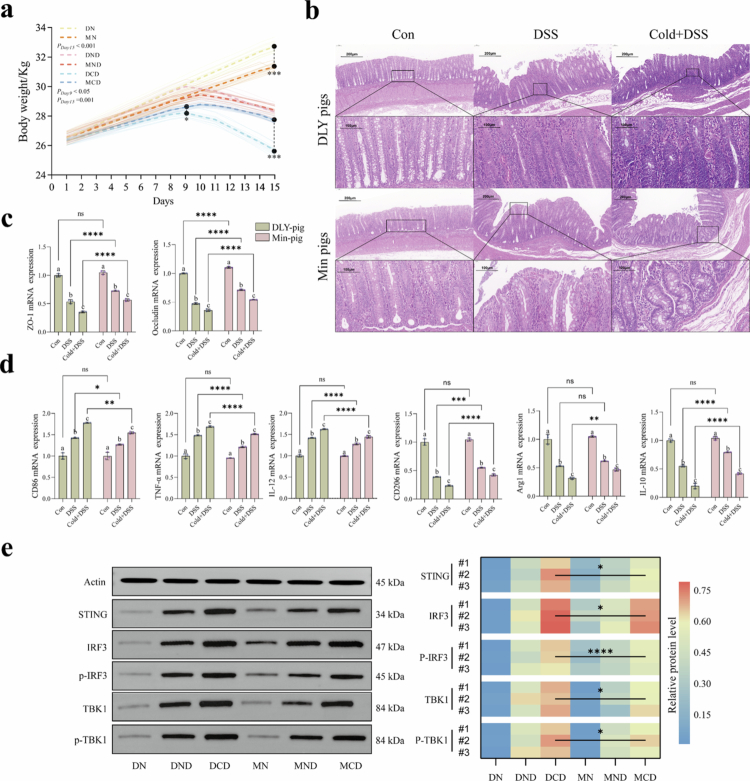
Min pigs maintain intestinal immune homeostasis by regulating the STING pathway under low temperature and DSS stimulation. (a) Trends in body weight of each group of pigs during 15 d of feeding. (b) Hematoxylin and eosin (H&E) staining of colonic tissues, illustrating histopathological changes. Scale bars: 100 and 200 µm. (c, d) Quantitative real‐time PCR (qRT-PCR) analysis of the mRNA expression levels of ZO-1, Occludin, CD206, Arg1, IL-10, CD86, TNF-*α*, and IL-1β in porcine intestines (*n* = 3). (e) Immunoblot analysis of STING, phosphorylated IRF3, total IRF3, phosphorylated TBK1, total TBK1, and actin (*n* = 3). Data were analyzed using two‐way ANOVA and are presented as the mean ± standard deviation from three biological replicates. *P < 0.017, **P < 0.01, ***P < 0.001, and ****P < 0.0001. a, b, and c represent the within-group differences between DLY pigs and Min pigs (*P* < 0.017).

Min pig is a local pig breed in Northeast China that is characterized by its ability to maintain immune homeostasis in a cold environment. To investigate the intrinsic mechanism of the immune characteristics of Min pigs, we conducted experiments using two pig breeds, Min pigs and DLY pigs, as cold temperature models, and the detailed grouping and steps are shown in Figure S1. We found that DSS-induced colitis caused significant differences in body weight changes in the two pig breeds at the beginning of the modeling period, but these differences disappeared at the end. However, hypothermic feeding changed this effect, with Min pigs losing less weight under hypothermic combined DSS stimulation compared to DLY pigs (Figure 1a). As observed by hematoxylin and eosin (H&E) staining of colon tissue sections, Min pigs and DLY pigs showed similar inflammatory responses to DSS-induced colitis under room temperature rearing conditions with no significant differences. However, Min pigs showed better immunity when reared at low temperatures. Specifically, the intestines did not show a reduction or disappearance of cup cells, and there was less infiltration of inflammatory cells and a more intact intestinal and crypt structure in the colons of Min pigs compared to DLY pigs (Figure 1b). The qRT-PCR results showed significantly higher expression levels of ZO-1 and Occludin in Min pigs compared to DLY pigs under both normothermic and low-temperature conditions (Figure 1c). These findings indicate that Min pigs maintain more robust intestinal barrier function, which is essential for preventing pathogen invasion and controlling inflammation in response to stress.^13^^,^^14^ These observations suggest that, at low temperatures, Min pigs exhibit superior inflammatory immunity to DLY pigs, but not at room temperature. However, Min pigs can maintain relatively good intestinal integrity in the face of DSS-induced colonic inflammation at both low and room temperatures, which may be the basis for Min pigs to retain good immunocompetence at low temperatures.

### Differential polarization and STING pathway activation in porcine macrophages at low temperature

To investigate the underlying mechanisms by which Min pigs show better disease resistance than DLY pigs under cold conditions, we analyzed the mRNA expression levels of M1 and M2 macrophage-associated cytokines in the colonic tissues of Min pigs and DLY pigs. Under DSS stimulation, M1 macrophage-associated cytokines were significantly increased and M2 macrophage-associated factors were significantly decreased in the colonic tissues of both breeds of pigs. Moreover, the combination of low temperature and DSS stimulation exacerbated this situation. Nevertheless, regardless of the stimulus conditions, Min pigs consistently displayed significantly higher levels of M2-associated cytokines (CD206, IL-10, and Arg1) and lower levels of M1-associated cytokines (CD86, TNF-*α,* and IL-1β) compared to DLY pigs (Figure 1d,e). These findings suggest that the superior disease resistance of Min pigs in low-temperature environments may be attributed to their ability to maintain effective polarization of M2 macrophages during intestinal inflammation,^15^ thereby promoting anti-inflammatory responses and tissue repair. Therefore, the unique macrophage polarization strategy in the colon of Min pigs may contribute significantly to alleviating excessive inflammation and maintaining intestinal homeostasis under stressful conditions.

Because STING activation is closely related to macrophage polarization homeostasis, to further investigate the potential reasons for the differences in intestinal macrophage polarization between Min pigs and DLY pigs in DSS-induced colitis in cold environments, we performed protein blotting analyses of the relevant proteins in the STING pathway in the colonic tissues of the two groups of pigs. Specifically, although the level of STING pathway activation was significantly elevated in both heat pig breeds, the elevated level was significantly lower in Min pigs than in DLY pigs (Figure 1e). In contrast, the high activation of STING-IRF3-TBK1 in the colon of DLY pigs may be due to an overactivation of an abnormal immune response. The overactivation of STING would mediate the polarization of M1 macrophages to enhance the inflammatory response and disrupt the normal immune homeostasis of the organism.^16^ This differential activation of the STING pathway may help explain the ability of Min pigs to maintain intestinal immune homeostasis and avoid the deleterious effects of hyperinflammation under stress conditions.^17^^,^^18^ These data reveal that Min pigs may have a mechanism that prevents overactivation of the STING pathway. Interestingly, this mechanism seems to be associated with M2 macrophage polarization in the gut of Min pigs, allowing the gut to maintain good immune homeostasis under stress conditions.

### Remodeling of the intestinal flora in Min pigs challenged by low temperature and DSS

**Figure 2.**
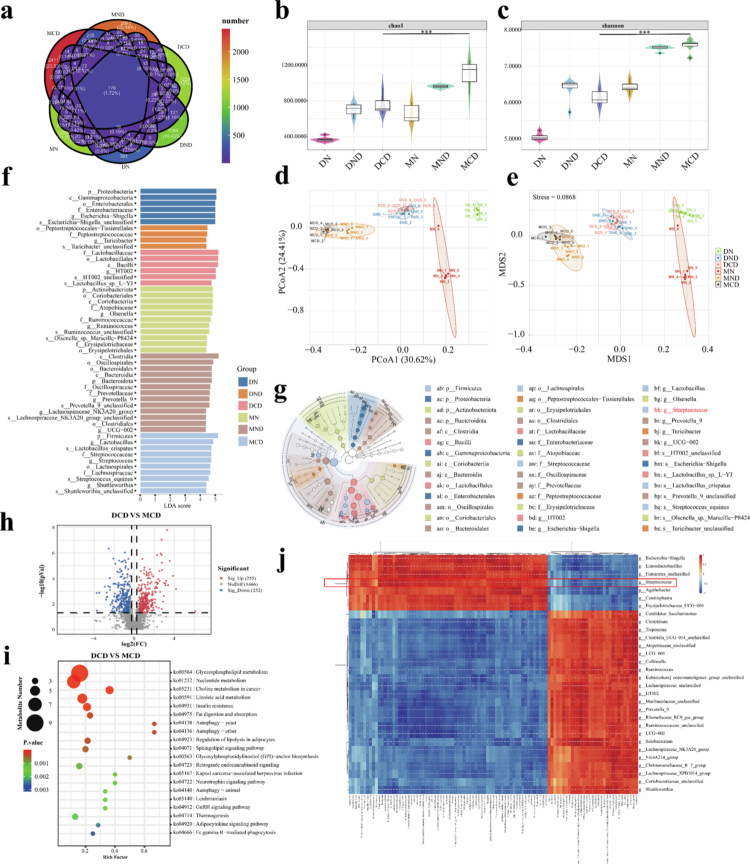
Significant differences in microorganisms and metabolites in the cecum contents of Min pigs and DLY pigs. (a) Differences in microbial abundance in the cecal contents of Min pigs and DLY pigs, especially breed-specific unique microbes, were analyzed by amplicon sequence variation (ASV) following a combination of DSS and low-temperature stimulation. Venn diagrams show the ASV overlap between the two groups, where each circle represents a subgroup, and the overlapping area indicates the number of ASVs shared between the groups. Non-overlapping areas indicate ASVs unique to each group. (b, c) Alpha diversity analysis comparing Chao1 and Shannon indices observed between experimental groups (*n* = 6 samples/group). (d) Beta diversity analysis including principal coordinate analysis (PCoA) of gut microbial composition for each experimental group (*n* = 6 samples/group). (e) Non-metric multidimensional scaling (NMDS) analysis (*n* = 6 samples/group) (stress = 0.0868). (f) Histogram of the distribution of LDA scores using linear discriminant analysis of effect size (LEfSe) analysis of differences in microbial abundance (*n* = 6 samples/group). (g) Branching diagram of the evolution of the dominant microorganisms in the pig intestine in each experimental group. (h) Volcano plots of differential metabolic pathways in the intestines of DCD and MCD pigs. (i) Enrichment fraction plots of metabolic pathways in the intestines of DCD and MCD pigs. (j) Plot of pathway enrichment scores between DCD and MCD pigs showing species and metabolite differences. Spearman correlations were calculated, and the colors represent the strength and direction of the correlation (red for positive correlation and blue for negative correlation). Darker colors indicate stronger correlations. *P < 0.017, **P < 0.01, and ***P < 0.001.

Hypothermia and DSS cause dramatic changes in microorganisms along with inflammation in the animal's gut, while indirectly affect STING activation.^19-21^ To elucidate the intrinsic relationship between preventing overactivation of STING pathways in the gut of Min pigs and changes in gut microbiology, we performed amplicon sequencing and metabolomics analyses of cecum contents from two pigs to elucidate any potential compositional differences. We quantified the total number of unique microbial sequences present in the cecum contents by amplicon sequence variant (ASV) analysis. In the graphs shown, MCD (2411 ASV) exhibited a higher number of ASVs than MND (2063 ASV) and DCD (1229 ASV). The DCD pigs also showed a higher number of ASVs compared to the DND (1069 ASVs) (Figure 2a). This difference in microbial richness suggests that Min pigs have a more diverse and unique gut microbial composition. We also calculated Alpha diversity using Chao1, Shannon (Figure 2b,c), Goods_coverage, Observe_species, and Simpson (Figure S2a–c) index to assess differences in gut microbial diversity between experimental pig groups. We observed that both Min pigs and DLY pigs exhibited changes in Alpha diversity when receiving DSS alone or under cold conditions. However, there was a significant difference in Alpha diversity between DCD and MCD, and Alpha diversity was higher in MCD. The gut microbial composition of both pigs in the normothermic attack groups (DND and MND) and the cryoattack groups (DCD and MCD) changed drastically, and DND vs DCD, MND vs MCD, and DCD vs MCD showed significant differences in Beta diversity (Figure S3), a measure of compositional similarity, as measured by the Bray‒Curtis distance in the nonmetric multidimensional scaling analysis (NMDS) with stress values less than 0.1 (stress = 0.0868) (Figure 2d,e).

To further resolve microbial differences between individuals in the control and experimental contexts of the two species, we assessed the taxonomic composition of the colonic microbiota. Compared with their respective room temperature control groups (DN, MN) and room temperature DSS groups (DND, MND), the cold stress combined with DSS group (especially DCD) showed increased relative abundance of potential opportunistic pathogens such as *Escherichia–Shigella*, *Clostridium sensu stricto 1*, and *Desulfovibrio*. These bacterial genera are frequently reported to be associated with intestinal inflammation and impaired barrier function, suggesting the disruption of intestinal homeostasis. Compared to the control group, the DCD group exhibited a downward trend in the relative abundance of probiotic or SCFA-producing bacteria such as *Lactobacillus*, *Subdoligranulum*, *Faecalibacterium*, *Butyricicoccus*, and *Blautia*. This finding indicates damage to the protective gut microbiota, supporting the biological basis that low temperatures increase the susceptibility of the host to inflammatory responses (Figure S4a). Comparing MCD and DCD groups, although both groups exhibited dysbiosis, the MCD group presented a higher proportion of probiotic bacteria and showed a relatively smaller increase in pathogenic bacteria (the Min pigs exhibited less microbial disturbance under cold intervention than the DLY pigs). This observation aligns with the physiological phenotype observed in our experiments, where Min pigs exhibited milder tissue inflammation and stronger immune homeostasis under cold stress. As shown in Figure 2f, we performed linear discriminant analysis effect size (LEfSe) analyses according to breed and different experimental contexts. In DCD, linear discriminant analysis scores were highest for *Bacilli*, *Lactobacillales*, *Lactobacillaceae*, and *Lactobacillus*, whereas in MCD, the highest LDA scores were found in *Lachnospiraceales* and *Streptococcaceae*. *Lachnospiraceae,Lactobacillus*, *Streptococcus*, and *Streptococcus*. In addition, we observed phylogenetic relationships of differentially abundant taxa from the evolutionary branching diagrams analyzed by LEfSe (Figure 2g). The gut microbes from DCD differed from those of MCD at the phylum level, but the differences were particularly significant at the genus level, with higher abundance of *Lactobacillus* and *Streptococcus*. The results of our study demonstrated that the intestinal tract of the MCD exhibited a higher abundance of beneficial bacterial species compared to pigs in the DCD (Figure S4b). Among these bacteria, *Lactobacillus* and *Streptococcus* (Figure S4c) have been identified as potential contributors to immune homeostasis maintenance.^22-24^

### *S. hyointestinalis* regulates host intestinal immune homeostasis

Gut microbes can have positive effects on the host, but these effects are usually indirect through metabolites.^25^^,^^26^ We analyzed differential metabolic pathways between DCD and MCD to identify microorganisms that may contribute to immunocompetence in Min pigs under stressful conditions (Figure S5). A total of 255 metabolic pathways were upregulated, and 1,466 pathways were downregulated in MCD compared to DCD (Figure 2h,i; Figure S6 a,b). Most of the quantitatively upregulated pathways were related to glycerophospholipid metabolism and nucleotide metabolism, but autophagy-related signaling pathways were the most enriched. To investigate the relationship between the differential microorganisms and metabolites, we performed Spearman correlation analysis between the differential species and metabolites. The results showed that several major metabolites were positively correlated with previously identified dominant microorganisms (*Lactobacillus* and *Streptococcus*). These metabolites (e.g., short-chain fatty acids,^27^ LysoPE (15:0/0:0),^28^ and 3-hydroxyoctadecanoic acid^29^) are commonly involved in the regulation of intestinal immune homeostasis and maintenance of gut health (Figure 2j; Figure S6c). Notably, some of these metabolites are associated with the production of EVs by bacteria. For example, LysoPE (15:0/0:0), a phospholipid amide that plays a crucial role in cell membrane structure and function, is an essential component of EVs.^30^^,^^31^ Similarly, 1-myristoyl-2-hydroxy-sn-glycero-3-phosphoethanolamine is a phospholipid derivative that is extensively involved in EVs formation and regulates vesicle release and content transfer by affecting the cell membrane structure.^32^^,^^33^ In addition, 9,12-octadecadiynoic acid, a long-chain unsaturated fatty acids, contributes to cell membrane fluidity and aids in the formation of EVs and intercellular communication.^34^ Extracellular vesicles, as macromolecular metabolites secreted by bacteria, have been shown to be involved in immunomodulatory processes.^35^^,^^36^

**Figure 3.**
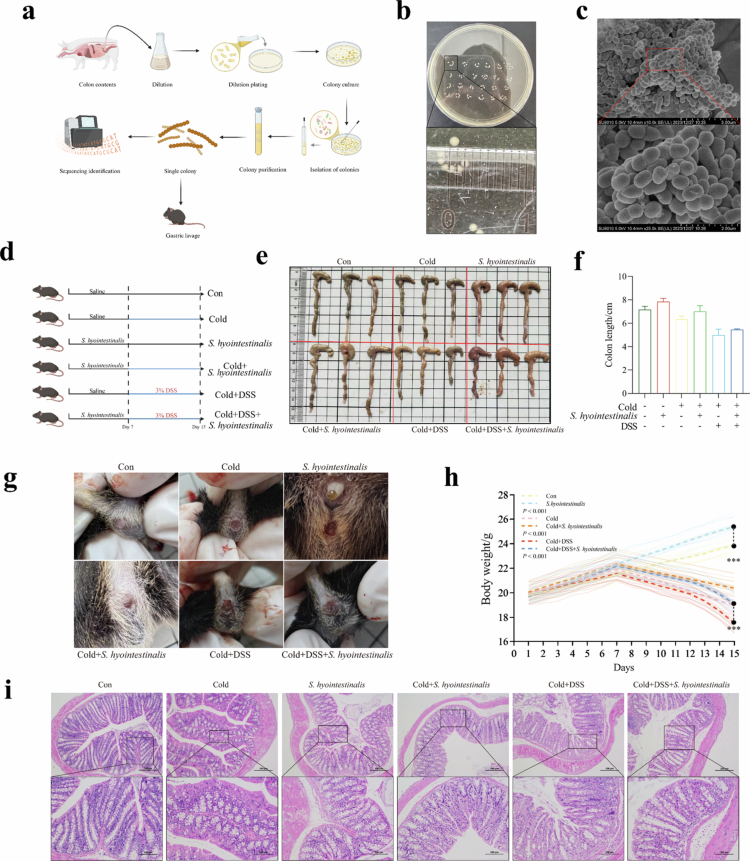
*S. hyointestinalis* alleviates low temperatures and DSS-induced colonic inflammation. (a) Schematic diagram of the isolation and identification of characteristic microorganisms from the intestinal tract of Min pigs and in vivo validation test. (b) Colony morphology of *S. hyointestinalis* strains. (c) Morphological characteristics of *S. hyointestinalis* under a scanning electron microscope. (d) Experimental design for investigating the role of *S. hyointestinalis* in colitis. (e) Photographs of representative colon lengths of the mice in each group (*n* = 3). (f) Statistical analysis of mouse colon length. (g) Perianal occult blood from each group of mice after 15 d of experimental feeding. (h) Trends in body weight of each group of mice during 15 d of feeding (*n* = 12). (i) H&E staining of colon tissue, showing histopathological changes in characteristic sections. Scale bars: 100 and 200 µm. Data were analyzed using two‐way ANOVA and are presented as the mean ± standard deviation from three biological replicates. *P < 0.017, **P < 0.01, ***P < 0.001, and ****P < 0.0001.

Based on the above studies, we successfully isolated highly EVs-producing anaerobic strains from the colonic contents of Min pigs by the dilution smear method and identified them as *S. hyointestinalis* by 16S rRNA sequencing (Figure 3a). *S. hyointestinalis* was cultured anaerobically in fastidious anaerobe agar (FAA) for 24 h. A single milky white colony was formed, which was smooth, with clear edges, and had a diameter of approximately 1 mm (Figure 3b). To further characterize *S. hyointestinalis*, we examined its morphology using a scanning electron microscope, which showed that the bacterium was spherical in form and was arranged in short chains or pairs (Figure 3c). To investigate the potential probiotic effects of *S. hyointestinalis* on the intestinal tract, we verified the effects of *S. hyointestinalis* on C57bl/6 mice subjected to DSS under cold conditions by tube feeding. Mice stimulated with DSS at low temperatures showed blood in the stool and large amounts of blood residues around the anus, but this effect was effectively mitigated by mice pre-administered with *S. hyointestinalis* tube feedings (Figure 3g). Additionally, *S. hyointestinalis* also ameliorates the elevation of serum cortisol (Figure S7b), weight loss (Figure 3h), colon shortening, and hemorrhage ​​​​​(Figure 3e,f) in mice subjected to cold stress or combined cold and DSS stimulation. We observed no significant difference in the body weights of the mice in all six experimental groups from day 1 to day 7. However, from day 8, the body weight of *S. hyointestinalis* mice was significantly higher than that of Con mice (*P* < 0.017). From day 12, the body weights of Cold mice were significantly different from those of Cold+*S. hyointestinalis* mice (*P* < 0.017), with Cold+*S. hyointestinalis* mice showing less weight loss. Starting from day 11, the decrease in body weight of Cold+DSS mice was smaller than that of Cold+DSS+*S. hyointestinalis* mice (*P* < 0.017), and the difference gradually increased from day 13 to day 15, with the most significant difference occurring on day 15 (*P* < 0.0005). H&E staining showed that the colonic mucosa of Cold+DSS mice had severe H&E staining showed that the colonic mucosa of Cold+DSS mice had severe inflammatory changes, including crypt shedding, abscess formation and inflammatory cell infiltration (Figure 3i). Although Cold mice showed no colon damage, they also had high levels of inflammatory cell infiltrations. In contrast, mice that were injected intragastrically with *S. hyointestinalis* showed reduced inflammatory cell infiltration, especially in Cold+DSS+*S. hyointestinalis* mice, where the colon cup cells remained relatively intact and the intestinal epithelial damage was less severe compared with Cold+DSS mice.

### *S. hyointestinalis* modulates colonic STING pathway activation and macrophage polarization

**Figure 4.**
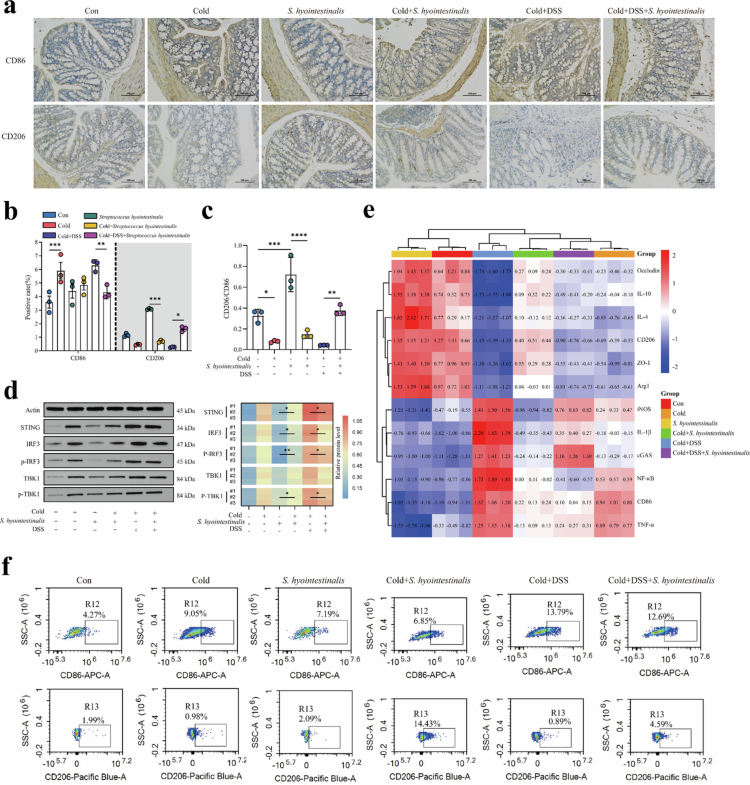
*S. hyointestinalis* promotes M2 macrophage polarization by inhibiting STING pathway hyperactivation. (a) Immunohistochemical staining of CD86 and CD206 in colon tissues. Representative images of immunohistochemical staining of CD86 (M1 macrophage marker) and CD206 (M2 macrophage marker) in colon sections from different experimental groups of mice. (b) Quantification of CD86 and CD206 immunohistochemical staining. The bar graphs show the relative intensity of CD86 and CD206 staining in colon tissue (*n* = 3). (c) Ratio of the relative intensities of CD206 and CD86 staining in immunohistochemical staining. (d) Immunoblot analysis of STING, phosphorylated IRF3, total IRF3, phosphorylated TBK1, total TBK1, and actin (*n* = 3). (e) Expression heat map based on the mRNA expression levels of ZO-1, Occludin, CD86, TNF-*α*, iNOS, IL-1β, CD206, Arg1, IL-4, IL-10, NF-κB, and cGAS in the mouse colon. The mRNA expression levels were analyzed by quantitative real-time PCR (qRT-PCR). (f) Flow cytometry analysis of macrophage polarization markers. Flow cytometry plots show the percentage of CD86 (M1 macrophage marker) and CD206 (M2 macrophage marker) cells in colon samples (*n* = 3). Data were analyzed using one-way ANOVA and are expressed as mean ±  standard deviation of three biological replicates. *P < 0.017, **P < 0.01, ***P < 0.001, and ****P < 0.0001.

Macrophages usually play a key role in the intestinal immune response.^37^^,^^38^ Our experimental data indicate that *S. hyointestinalis* has a significant mitigating effect on DSS-induced colitis in both normothermic and hypothermic rearing. This prompted us to explore the effect of *S. hyointestinalis* on the host immune response to colitis induced by low temperature and DSS using a mouse model of colitis reared at low temperature. We first crudely measured changes in the number of M1 macrophages (CD86) and M2 macrophages (CD206) in the experimental mouse colon by immunohistochemistry (Figure 4a). Compared with the control mice, both Cold mice and Cold+DSS mice showed cellular polarization dominated by M1 macrophages, specifically an increase in M1 macrophages and a decrease in M2 macrophages, which was more pronounced in the Cold+DSS mice. However, compared to both stimulation groups, mice that were pre-administered with *S. hyointestinalis* gavage had a significant remission phenomenon in the same situation, resulting in a decrease in M1 macrophages, which dominate acute inflammation, but an increase in M2 macrophages, which are normally inhibited from polarization (Figure 4b,c). To validate the previous work, we measured the expression of STING-IRF3-TBK1 axis proteins in mouse colon tissues and obtained results similar to those of previous experiments. STING activation is usually associated with the host because of the immune response, but the massive expression of STING proteins when the host is exposed to cold environments and DSS stimuli implies an over-activation of immune signaling pathways, a phenomenon that usually exacerbates inflammation. *S. hyointestinalis* well-inhibited the overactivation of the STING-IRF3-TBK1 axis due to dual stimulation, and was the reduction of STING, IRF3, TBK1, *p*-IRF3, and *p*-TBK1 protein expression. The results revealed an interesting finding: Interestingly, when the STING was overactivated, M1 macrophages were heavily polarized. In contrast, inhibition of the STING led to a decrease in the number of M1 macrophages and an increase in M2 macrophages (Figure 4d).

To further explore the effects of STING activation on colitis and macrophage polarization, we determined the mRNA expression of a few cytokines associated with macrophage polarization and inflammation. Compared with the stimulated group (Cold and Cold+DSS mice), the overall trend of tight junction proteins (ZO-1 and Occludin) in the colon of the relieved group (Cold+*S. hyointestinalis* and Cold+DSS+*S. hyointestinalis* mice) was effectively controlled, although it was still significantly reduced. Gene expression of M1 macrophage surface markers (CD86), functional markers (iNOS and TNF-*α*), and inflammatory factors (IL-1β) was significantly lower in the remission group than in the stimulation group, whereas the expression of M2 macrophage-associated genes (CD206, Arg1, IL-4, and IL-10) expression was significantly higher than in the stimulation group. We next focused on the expression of key genes in the upstream and downstream pathways of the STING. cGAS, a key sense receptor that initiates the activation of the STING, did not decrease in expression because of *S. hyointestinalis* intervention, whereas the expression of NF-κB was significantly reduced. This suggests that *S. hyointestinalis* did not affect the process of STING protein activation by cGAS throughout the upstream and downstream pathways involved in the activation of the STING, but rather directly affected the process of STING activation, thereby achieving the inhibition of its overactivation (Figure 4e). To assess the effect of the STING on macrophage polarization more accurately, we accurately calculated the number of M1 versus M2 macrophages in the colonic tissues by flow cytometry, and the number of M2 macrophages was significantly increased in the remission group than in the control group (Figure 4f). This further suggests that *S. hyointestinalis* alleviates low temperature and DSS-induced colonic inflammation by inhibiting the overactivation of the STING-IRF3-TBK1 axis.

### Host response to *S. hyointestinalis* EVs in colitis

**Figure 5.**
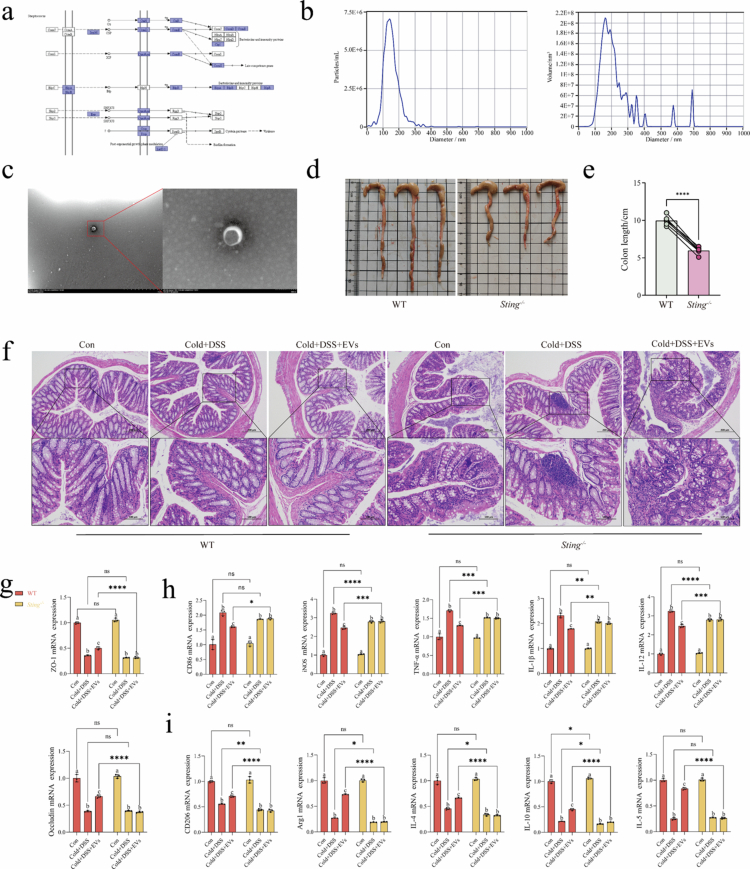
*S. hyointestinalis* EVs relieves colitis. (a) Excerpt of the KEGG pathway map from the whole-genome analysis results of *S. suis*. (b) Characterization of the EV concentration and particle size of *S. suis* by nanoparticle tracking analysis (NTA). (c) Morphological characterization and particle size analysis of *S. suis* by projection scanning electron microscopy. (d) Comparison of colon length in WT and *Sting*^-/-^ mice that received EV gavage from days 1–12 and were fed at low temperature (4–8 °C) with 3% DSS drinking water from days 8–12 (*n* = 3). (e) Quantification of colon length in WT and *Sting*^-/-^ mice (*n* = 3). (f) Histological analysis of colon sections stained with H&E from WT and *Sting*^-/-^ mice. Groups: Con (saline gavage for 1‒12 d), Cold+DSS (saline gavage for 1‒12 d, cold feeding at 4‒8 °C and 3% DSS drinking water for days 8‒12), Cold+DSS+EVs (EV gavage for 1‒12 d, cold feeding at 4‒8 °C and 3% DSS drinking water for days 8‒12). Scale bars: 100 and 200 µm. (g–i) qRT-PCR analysis of the mRNA expression levels of tight junction proteins, M1 macrophage-related cytokines, and M2 macrophage-related cytokines in the porcine colon (*n* = 3). Data were analyzed by two-way ANOVA and are presented as the mean ± SD of three biological replicates. *P < 0.017, **P < 0.01, ***P < 0.001, and ****P < 0.0001. a, b, and c represent intra-group differences between the WT and *Sting*^-/-^ groups (*P* < 0.017).

To determine the specific mechanism by which *S. hyointestinalis* suppresses STING-IRF3-TBK1 axis hyperactivation, we evaluated the potential role of the extracellular vesicles it produces on colitis. In addition, we investigated the intrinsic association of the *S. hyointestinalis* EVs–STING–macrophage axis through a colitis model in cryopreserved *Sting*^*-/-*^ mice. In a previous study, we found that *S. hyointestinalis* possesses excellent extracellular vesicle-producing ability (Figure 5a). This prompted us to comprehensively analyze all aspects of its ability through genome-wide techniques. *S. hyointestinalis* possesses genes related to bacteriocin and immune protein secretion, and EVs are their key vectors;^39^^,^^40^ therefore, we explored the role of *S. hyointestinalis* EVs in colitis. First, we extracted and characterized the EVs of *S. hyointestinalis* using differential centrifugation^41^ and revealed that the size of *S. hyointestinalis* EVs was 191.3 nm and was a well-defined sphere with clear edges under transmission electron microscopy (Figure 5b,c).

Next, we verified the modulatory effects of *S. hyointestinalis* EVs on the STING–IRF3–TBK1 axis and their potential to alleviate colitis by co-stimulating WT mice and *Sting*^*-/-*^ mice, which had been pre-gavaged with EVs, with both hypothermia and DSS. We found that *Sting*^*-/-*^ mice did not have reduced colon length shortening or bleeding symptoms compared with WT mice, despite the fact that both groups of mice had been pregavaged with EVs (Figure 5d,e). In addition, H&E staining showed intense and pronounced inflammation, including crypt rupture, abscess formation, and extensive inflammatory infiltration in WT-Cold+DSS mice. However, in WT-Cold+DSS+EVs mice, the inflammation was significantly alleviated to some extent; although the crypts became shorter, they remained, and there was no extensive inflammatory cell infiltration. In contrast, *Sting*^*-/-*^-Con mice exhibited mild inflammation in the colon, which was markedly exacerbated by combined stimulation of Cold+DSS. This included the loss of crypts, massive reduction of cup cells, and massive aggregation and infiltration of inflammatory cells, which were not significantly alleviated by EVs treatment (Figure 5f). The qRT-PCR results showed that EVs effectively maintained the integrity of the colon in WT mice and promoted the expression of tight junction proteins. In addition, EVs promoted the polarization of M2 macrophages (CD206 and Arg1), inhibited the polarization of M1 (CD86 and iNOS) macrophages, and significantly reduced the expression of inflammatory factors (TNF-*α*, IL-1β, IL-12, IL-4, IL-10, and IL-5). In contrast, no similar effect was observed in *Sting*^*-/-*^ mice, where M1 macrophages were significantly polarized, M2 polarization was inhibited, and inflammatory factor expression was elevated in severe colitis induced by DSS at low temperature. Importantly, EVs intervention did not ameliorate these conditions in *Sting*^*-/-*^ mice (Figure 5g–i). These findings suggest that the alleviating effects of EVs on colitis and the modulation of macrophage polarization homeostasis may be dependent on the STING–IRF3–TBK1 axis.

### *S. hyointestinalis* EVs target STING pathway overactivation to regulate macrophage polarization

**Figure 6.**
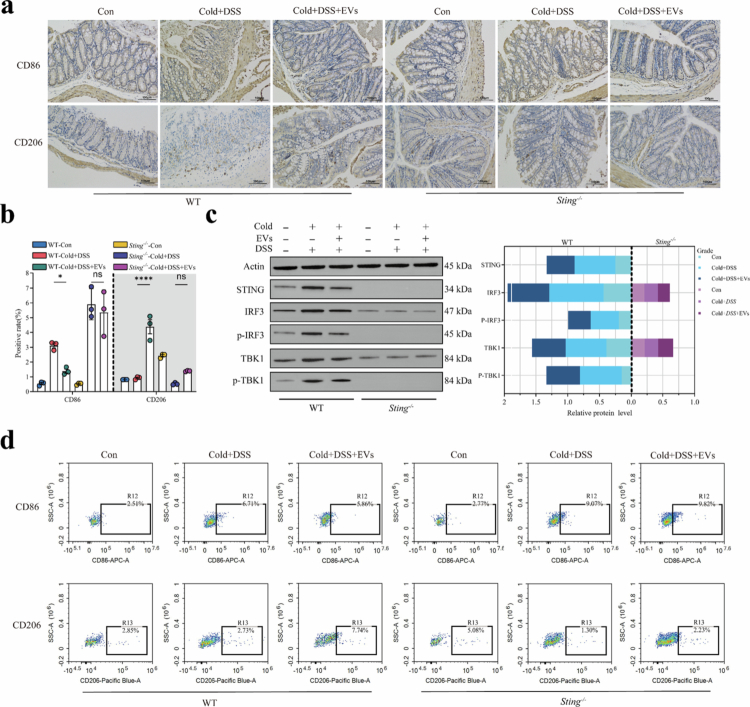
Immunologic role of *S. hyointestinalis* EVs. (a) Immunohistochemical staining of CD86 and CD206 in colon tissues of WT and *Sting*^-/-^ mice. (b) Analysis of the positive rate of immunohistochemical staining of CD86 and CD206 in mouse colon tissues (*n* = 3). (c) Relative intensity ratio of CD206 and CD86 staining via immunohistochemical staining (*n* = 3). (c) Immunoblot analysis of STING, phosphorylated IRF3, total IRF3, phosphorylated TBK1, total TBK1, and actin in the colon tissues of WT and *Sting*^-/-^ mice. (d) Flow cytometric analysis of macrophage polarization markers by gating strategy. Flow cytometric graphs show the percentage of CD86 (M1 macrophage marker) and CD206 (M2 macrophage marker) cells in colon samples. Data were analyzed using two-way ANOVA and are expressed as the mean ± SD of three biological replicates. *P < 0.017, **P < 0.01, ***P < 0.001, and ****P < 0.0001.

EVs serve as carriers for bacteria to transfer bioactive molecules to recipient cells, suggesting that certain bacterial biological functions may be associated with or originate from these EVs.^42-44^ *S. hyointestinalis* EVs alleviate DSS-induced colitis exacerbated by hypothermia and modulate macrophage polarization homeostasis, but the exact mechanism is not yet clear. To explore this, we examined the effects of *S. hyointestinalis* EVs on macrophage polarization and highlighted the relevance of the STING–IRF3–TBK1 axis in this process. Immunohistochemical staining of mouse colon tissues showed that Cold+DSS stimulation resulted in a significant increase in M1 macrophage (CD86) positivity in WT and *Sting*^*-/-*^ mice. Meanwhile, the CD86 positivity rate was significantly higher in WT mice compared with *Sting*^*-/-*^ mice. On the other hand, M2 macrophages (CD206) exhibited significant inhibition of polarization, with similar levels of CD206 positivity in WT and *Sting*^*-/-*^ mice. However, EVs reversed this effect of hypothermic DSS in WT mice, modulating the balance of macrophage polarization in the colon and polarizing them toward an inflammation-suppressive and repair phenotype, an effect not observed in *Sting*^*-/-*^ mice. (Figure 6a-c). Furthermore, immunoprotein traces showed that EVs regulated the STING–IRF3–TBK1 axis in a manner similar to *S. hyointestinalis* in WT mice, but not in *Sting*^*-/-*^ mice (Figure 6d). Additionally, flow cytometry analysis of macrophage polarization in the mouse colon showed that EVs alleviated M1 macrophage hyperpolarization and inhibited M2 macrophage polarization in WT mice. This effect was not observed in *Sting*^*-/-*^ mice, in which M1 macrophage hyperpolarization was exacerbated (Figure 6e). The results of the above data suggest that *S. hyointestinalis* dynamically regulates STING–IRF3–TBK1 axis activation through the production of EVs to balance the macrophage polarization process in the colon, which in turn regulates intestinal immune homeostasis and maintains intestinal health. Although developmental differences in *Sting*^*⁻/⁻*^ mice cannot be fully excluded, the lack of response to canonical STING agonists and the absence of compensatory activation of the TLR4 or inflammasome pathways strongly support a STING-dependent mechanism (Figure S7c,d).

### Macrophage response to *S. hyointestinalis* EVs

Through multiple intervention assays and concentration-gradient experiments, we further confirmed the specificity of *S. hyointestinalis*-derived EVs in mediating the observed effects. Notably, treatment of EVs with phosphodiesterase (PDEs) markedly attenuated both the suppression of STING activation and the induction of M2 polarization in LPS-stimulated 3D4/21 cells (Figure S8b, c). Moreover, gradient co-culture experiments revealed that STING signaling was inhibited at an early stage (1 h) following EV exposure, preceding the onset of macrophage polarization (Figure S8d, e). We also found that, compared to EVs derived from other microbial sources, *S. hyointestinalis* EVs effectively bind to the STING protein, and this binding is competitive with c-di-AMP (Figure S10a–c). In a Transwell co-culture experiment involving polarized macrophages and IPEC-J2 cells (Figure S10d), we found that M2 macrophages significantly promoted IPEC-J2 cell migration, whereas M1 macrophages markedly inhibited IPEC-J2 cell re-epithelialization (Figure S10e, f). We subsequently fluorescently labeled EVs using a kit and stimulated macrophages with labeled EVs for 3 h to investigate whether EVs regulate macrophage signaling pathways by entering cells. The results demonstrated that EVs could enter macrophages and localize to the nucleus (Figure S11a, b). Immunoblotting revealed that EVs suppressed excessive activation of the cellular STING pathway, mirroring observations in mice. However, this suppression disappeared when the expression of the cellular STING gene was completely inhibited using H151 (Figure S11c, d). Similarly, flow cytometry analysis revealed reduced M1 macrophage polarization and increased M2 macrophage polarization in the EV-treated group of LPS-stimulated macrophages. However, this inhibitory effect of EVs was lost upon complete inhibition of the cellular STING pathway using H151 (Figure S11e). These findings indicate that EVs can enter macrophages and inhibit excessive STING signaling pathway activation by binding to the STING protein. This prevents excessive M1 macrophage polarization in response to external stimuli, thereby promoting macrophage polarization toward pro-inflammatory cells. Conversely, this indirectly promotes M2 macrophage polarization and enhances macrophage-mediated inflammation suppression.

## Discussion

The interplay between environmental stresses, the gut microbiota, and host immunity represents a frontier for understanding the pathogenesis of inflammation.^45^^,^^46^ Cold temperatures increase mitochondrial ROS production and lipid peroxidation, leading to the opening of the mitochondrial permeability transition pore,^47-50^ the release of mtDNA into the cytoplasm and its binding to cGAS-STING, and the facilitation of TBK1/IRF3-driven type I interferon and IL-1β transcription, which exacerbates intestinal inflammation.^51^^,^^52^ However, the mechanisms linking the regulatory effects of gut microbes on STING to the gut immune response process in animals resistant to cold-induced intestinal inflammation are not clear. In this study, we revealed the mitigating effect of a *S. hyointestinalis* and its EVs screened and isolated from the intestinal tract of a low temperature resistant species, Min pigs, on intestinal inflammation under low-temperature conditions. Mechanistically, the Min pigs-derived *S. hyointestinalis* targets and regulates the STING signaling pathway through the production of EVs, which in turn reshapes the balance of intestinal macrophage polarization to maintain intestinal immune homeostasis to ensure organismal health.

Furthermore, our study introduces a novel experimental framework by integrating environmental cold stress with the regulation of intestinal immune homeostasis. Previous research on microbial EVs and STING signaling has been conducted predominantly under normothermic conditions, with limited attention given to environmental stressors despite their significant physiological relevance in mammals and humans. We demonstrated that cold stress exacerbates STING-driven intestinal inflammation and disrupts the microbial composition, while EVs from *S. hyointestinalis* counteracts this effect by suppressing STING overactivation. This highlights an underexplored pathway linking environmental signals, microbial EV signaling, and the innate immune balance, providing a framework closer to real-world ecological and environmental conditions. In a study on the differences in colitis induced by cold stress combined with DSS in different pig breeds, we found that the cold-tolerant pig breeds, Min pigs, had better intestinal integrity and less intestinal inflammation in a low-temperature environment. Moreover, the level of M2 macrophage polarization in the intestines of Min pigs was not significantly suppressed by intestinal inflammation, which was different from that observed in the intestines of DLY pigs. In addition, this study revealed that hypothermia combined with DSS stimulation significantly activated the STING pathway in the colon of DLY pigs, as evidenced by the overexpression of STING proteins and abnormally elevated levels of downstream IRF3/TBK1 phosphorylation, whereas the activation of this pathway was significantly lower in Min pigs. Notably, the activation level of the STING pathway in the pig intestine was positively correlated with M1 macrophage polarization and negatively correlated with M2 macrophages. These findings suggest that some mechanism may regulate the activation of the STING pathway in the intestine of Min pigs and also explains that Min pigs reduced the susceptibility of Min pigs to DSS at low temperatures by suppressing the over-activation of STING through this mechanism.

Through 16S rRNA analysis, we observed that the relative abundance of dominant phyla – such as *Firmicutes* and *Bacteroidetes* – in the intestinal tracts of Min pigs shifted markedly under low-temperature conditions. Moreover, several putative probiotic genera, including *Lactobacillus*, *Streptococcus*, and *Ruminococcus*, were relatively enriched. Further metabolomic profiling identified 255 upregulated metabolites – including LysoPE (15:0/0:0) and 3-hydroxyoctadecanoic acid – that accumulated in Min pig intestines under combined low-temperature and DSS stimulation. These differential metabolites participated primarily in steroid biosynthesis, glycerophospholipid metabolism, the cAMP signaling pathway, and neuroactive ligand–receptor interactions. KEGG enrichment analysis revealed significant overrepresentation of glycerophospholipid metabolism and the cAMP signaling pathway. Glycerophospholipids, as the principal constituents of cellular membranes, and their metabolites (e.g., LysoPE) influence immune cell signaling by regulating membrane fluidity. To explore microbe–metabolite relationships, Spearman correlation analysis demonstrated a significant positive association between *Streptococcus* abundance and LysoPE (15:0/0:0) levels in Min pigs. These findings suggest that *Streptococci* of the dominant genus, may be involved in regulating immune homeostasis through biofilm-related mechanisms. Microorganisms are one of the important players in regulating intestinal immune homeostasis, which prompted us to further analyze the effect of dominant microorganisms in the intestine of pigs to alleviate intestinal inflammation caused by low temperature. According to our results, the dominant intestinal microorganisms in Min pigs are *Streptococci*, so we screened and isolated Min pigs-derived microorganisms from the cecum contents of Min pigs reared in a cold environment. And we successfully screened Min pigs-derived *S. hyointestinalis*. Next, we verified the probiotic effect of *S. hyointestinalis* in a mouse model of hypothermia combined with DSS-induced colitis. We found that both weight loss and intestinal inflammation in mice caused by hypothermia combined with DSS stimulation were significantly alleviated under the intervention of *S. hyointestinalis*. Moreover, similar results to those of previous studies were demonstrated in terms of macrophage polarization and STING pathway activation. Specifically, *S. hyointestinalis* effectively mitigated STING activation through overactivation while promoting macrophage polarization toward the M2 phenotype.

Once thought to be cellular debris, EVs are now recognized as complex interorganism communication vectors.^53^ With the gradual deepening of the understanding of various EVs, the complexity of their vesicle biosynthesis, cargo loading, release pathways, targeting mechanisms, and vesicle processing has led to an expanding dimension of research in this field. While fungal EVs utilize STING to amplify inflammation (often to the detriment of the host),^54^^,^^55^ recommend that bacterial EVs choose the same pathway to enforce immune quiescence.^56-58^ Recent studies indicate that extracellular vesicles derived from different members of the gut microbiota may exert distinct regulatory effects on the cGAS‒STING axis.^59-61^ For instance, reports indicate that fungal-derived extracellular vesicles can deliver immunostimulatory nucleic acids, activate STING, and promote type I interferon responses, ultimately exacerbating intestinal inflammation.^62^ Conversely, extracellular vesicles from commensal or symbiotic bacteria have been demonstrated to maintain immune homeostasis, partly by suppressing overactivated innate immune signaling.^63^^,^^64^ Our findings further expand this concept by demonstrating that extracellular vesicles produced by the commensal bacterium *S. hyointestinalis*, which is enriched in Min pigs, suppress abnormal STING activation and promotes macrophage polarization toward the anti-inflammatory M2 phenotype. Unlike fungal EVs, which act primarily as STING agonists, *S. hyointestinalis* EVs contain cyclic diadenosine analogues that directly bind to STING, functioning as modulators to fine-tune rather than amplify STING activity. This comparison demonstrated that the immunological outcomes of EVs-STING interactions are highly dependent on the microbial origin of the vesicles. This finding underscores the dual role of STING as both an inflammatory sensor and a guardian of homeostasis within the host-microbiota-immune axis, highlighting its context-specific nature. Bacterial EVs carry pathogen-associated molecular patterns (PAMP), metabolites, and nucleic acids that modulate host immunity.^65^^,^^66^ Based on the ability of our screened Min pigs-derived *S. hyointestinalis* to produce EVs, we validated the mechanism of action of EVs by targeting the regulation of STING to maintain intestinal immune homeostasis in Sting^-/-^ mice. We found that EVs are indeed responsible for the probiotic effects of *S. hyointestinalis*. STING knockdown removed the remodeling of the polarization balance of mouse intestinal macrophages and the mitigating effects of EVs on intestinal inflammation. In further studies, it was clearly shown that the fluorescent labeling of EVs can be endocytosed by macrophages. And EVs alleviated the LPS-induced macrophage polarization toward the M1 phenotype, and this phenomenon disappeared under the interference of STING inhibitors.

Although this study focused on Min pigs, multiple lines of evidence suggest its potential translational application value. Multiple species of commensal *Streptococci* are common components of the human microbiome and are known to release exosomes to regulate host immune pathways.^67-69^ For example, EVs derived from commensal gut bacteria have recently been shown to carry cargoes that influence epithelial and immune cell responses in the gut and beyond.^70-72^ Moreover, a study specifically demonstrated that *S. salivarius* K12 produces EVs of 123 nm diameter which stimulate IL-1β and IL-8 production in a human monocytic cell line, indicating that human-associated streptococci indeed generate immunomodulatory EVs.^73^ The STING–TBK1–IRF3 signaling pathway exhibits high evolutionary conservation between pigs and humans, with key structural features of the adaptor protein STING, such as the cyclic-dinucleotide binding domain and the C-terminal tail (CTT) showing significant sequence and structural homology. For example, structural reviews of STING highlight that the ligand-binding domain (LBD) and the CTT are crucial for TBK1 recruitment and IRF3 activation in humans.^74^ One mechanistic study noted that although core components of the cGAS–STING pathway are conserved from bacteria to humans, vertebrate STING uniquely contains the CTT fragment that binds TBK1 and IRF3.^75^ Meanwhile, work in porcine macrophages showed that porcine IKKε (a TBK1-family kinase) is involved in STING-induced type I IFN expression, supporting functional conservation across species.^76^ Therefore, these conserved structural and functional characteristics support the possibility that exosomally derived exosomes from microbial sources may regulate human STING-mediated macrophage responses in a manner similar to that observed in this study.

## Conclusion

Our research demonstrates that EVs derived from *S. hyointestinalis* can target and suppress excessive STING signaling in the gut by binding to the STING protein, thereby promoting macrophage polarization toward the M2 phenotype and alleviating colitis. These findings reveal that microbe-derived EVs play crucial regulatory role in maintaining host immune homeostasis. Furthermore, our mechanistic analysis of the intervention trials indirectly confirms that EVs derived from *S. hyointestinalis* may contain cyclic dinucleotide analogues. These analogs further regulate the signaling pathway by binding to STING. These findings provide a clear molecular basis for EVs-mediated STING activity regulation and reveals a novel host–gut microbiota–immune axis regulatory mechanism. Notably, despite no change in cGAS transcription levels after EVs treatment, the finding that STING binds to EVs suggests that EVs primarily exert their effects through a cGAS-independent mechanism by directly regulating STING. This direct interaction provides a mechanistic explanation for how extracellular vesicles fine-tune downstream signaling pathways and promote macrophage M2 phenotype conversion. However, further studies are needed to determine whether other extracellular vesicle components also participate in regulating upstream or downstream signaling pathways of the STING signaling pathway and to comprehensively elucidate the breadth and depth of interactions between extracellular vesicles and host cells.

## Methods

### Animals

Min pigs and DLY pigs (Duroc × Landrace × Yorkshire, DLY pigs) weighing 25 ± 1 kg at the nursery stage were selected and divided into three experimental groups of six pigs of each breed: the normothermic group (DN group, MN group): pigs were kept at room temperature (20–22 °C) for 15 d without any stimulus. For the normothermic attack group (DND group, MND group): pigs were kept at room temperature (20–22 °C) for 15 d, and colitis was induced by giving drinking water containing 3% DSS from the 8th day. Hypothermic attack group (DCD group, MCD group): pigs were kept at room temperature (20–22 °C) for 7 d, and from day 8 onwards, they were subjected to hypothermia (4–8 °C) and given drinking water containing 3% DSS to induce colitis. Euthanasia was performed by electric shock at the end of the experiment. For the pig experiments, we selected groups of *n* = 3–6 due to ethical and logistical constraints in large animal studies. Post-hoc power calculations used the observed variance for the primary endpoint and assumed a two-tailed *α* = 0.05. Under these conditions, the study had ≥ 80% power to detect between-group differences of approximately 1.2–1.8 SD (equivalent to approximately 28%–45% change) (Note: For pigs weighing approximately 25 kg, the critical low temperature is typically approximately 15–18 °C, depending on the housing conditions and genetic background. As a pig breed with excellent cold tolerance in northeastern China, the Min pig thrives at ambient temperatures of 4–8 °C – well below the critical low – and has been widely used in previous studies to induce moderate to severe cold stress in pigs.^77-79^ Importantly, this temperature range reliably elicits physiological stress responses without causing hypothermia or overt disease, making it suitable for simulating chronic cold stress under controlled experimental conditions. Thus, we selected 4–8 °C as the cold exposure temperature to ensure the sustained activation of stress-related physiological pathways while minimizing impacts on animal welfare.

C57BL/6 mice (6-8 weeks old males, body weight 20.0 ± 2.5 g, purchased from Liaoning Changsheng Biotechnology Co., Ltd.) were selected and randomly divided into 6 groups, with 12 mice replicated in each group. The Con group: 1–15 d of room temperature (20–22 °C) rearing and saline gavage. Cold group: 1–7 d of room temperature rearing, 8–15 d of low-temperature (4–8 °C) rearing, and saline gavage throughout. *S. hyointestinalis* group: 1–15 d of room temperature rearing and bacterial liquid gavage. Cold+*S. hyointestinalis* group: 1–7 d of room temperature rearing, 8–15 d of low-temperature rearing and bacterial liquid gavage throughout. The Cold+DSS group, 1–7 d of room temperature rearing, saline gavage, and 3% DSS drinking water for 8–15 d of low-temperature rearing. Cold+DSS+*S. hyointestinalis* group: 1–7 d of room temperature rearing, 8–15 d of low-temperature rearing, 3% DSS drinking water, and gavage with bacterial solution throughout. Samples were taken by necropsy at the end of the experiment.

The experiment involved two strains of wild-type (WT) and Sting knockout (*Sting*^*-/-*^) mice (6–8-week-old males, body weight 20.0 ± 2.5 g) (purchased from Liaoning Changsheng Biotech Co., Ltd.). Three groups were set up for each of the two strains, with 12 mice replicated in each group: the Con group: room temperature rearing with saline gavage from days 1–15; the Cold+DSS group: room temperature rearing from days 1–7; low-temperature (4–-8 °C) rearing with 3% DSS drinking water and saline gavage from days 8–15; the Cold+DSS+EVs group: mice reared at room temperature from days 1–7; the low-temperature rearing with 3% DSS drinking water and saline gavage from days 8–15; the Cold+DSS+EVs group: mice reared at room temperature from days 1–7; and the low-temperature rearing with 3% DSS drinking water and saline gavage from days 8–15. For the Cold+DSS+EVs group: the mice were kept at room temperature for 1–7 d; on days 8–15, the mice were kept at low temperature, given 3% DSS in the drinking water, and gavaged with EVs dissolved in PBS throughout the experimental period. Samples were taken by necropsy at the end of the experiment. The mice were administered EVs by oral gavage at 1 × 10⁸ particles per gram of body weight, once daily. EVs were diluted in PBS and delivered in a final volume of 200  μL per mouse. Based on body weight, this corresponded to approximately (2.0–3.0) × 10⁹ particles per dose for 20–30 g mice.

### Screening of *S. hyointestinalis*

First, we analyzed the microorganisms with LDA scores greater than 4 in the intestines of pigs to identify the dominant microorganisms in the intestines of Min pigs with MCD. Then, based on the combined analysis of significantly different metabolites and dominant microorganisms in DCD vs MCD, we screened for dominant microorganisms that might regulate the immune homeostasis of Min pigs' intestines.

### EVs isolation and purification

Bacterial EVs were isolated from *S. hyointestinalis* as described above. Briefly, the bacteria were inoculated with one colony in FAA and incubated overnight under anaerobic conditions at 37 °C with constant shaking. The cell suspension was centrifuged at 1500 × g, and the resulting supernatant was centrifuged again at 5000 × g. The supernatant was then centrifuged again at 5000 × g. The supernatant was subsequently filtered through a 0.8 µm membrane and ultracentrifuged at 150,000 × g. The supernatant was then filtered through a 0.8 µm membrane. After discarding the supernatant, the EVs were suspended in PBS and the EV suspension was stored at −80 °C.

### Cell

All the macrophages were cultured in complete Roswell Park Memorial Institute (RPMI) 1640 medium supplemented with 10% FBS, penicillin and streptomycin (Pen+Strep) at 37 °C in 5% CO_2_. The following cell lines were used: 3D4/21 cell line (RRID: CVCL_0F14) (purchased from Fenghui Bio, Hunan, China). The porcine alveolar macrophage cell line 3D4/21 (RRID: CVCL_0F14) is derived from a certified cell bank and is registered in Cellosaurus.

3D4/21 (porcine alveolar macrophages) were purchased from Fenghui Bio, Hunan, China. Mycoplasma screening was performed using species-specific primers (ACACCATGGGAGCTGGTAAT) and (CTTCTTCGACTTCCAGACCCAAGGCAT) with Gold Mix (Tsingke Biotechnology Co., Ltd. TSE101, China). Positive and negative controls were included in each experiment. The cells were tested 48 h prior to experimentation; all the results were negative (see Supplementary Figure S11). No standardized STR testing panel exists for porcine cell line identification; thus, a multiplex species-specific qPCR system was employed for species identification and contamination assessment. Genomic DNA extraction was performed using the Genomic DNA Extraction Kit (Axygen MAG-T-GDNA-M US) following the manufacturer’s instructions. Multiplex PCR amplification and qPCR detection targeted species-specific genetic markers for pigs, bovines, dogs, rabbits, monkeys, and cats. Reactions were performed on an ABI 7500 real-time PCR instrument, with genomic DNA from each species serving as a positive control and a no-template control included in each run. The 3D4/21 cell sample was amplified with only pig-specific primers, confirming its origin as Sus scrofa cells and revealing no evidence of cross-species contamination. Representative amplification curves are shown in the Supplementary Table. S5, 6.

The stained EVs were co-cultured with macrophages for 3 h. After staining the macrophages with DAPI, the EVs were observed by laser confocal microscopy to determine whether the EVs could enter the macrophages.

First, 500 ng of LPS, 1 × 10⁸ particles/mL of EVs, 5 μm of H-151, 500 ng of LPS and 1 × 10⁸ particles/mL of EVs, 500 ng of LPS and 5 μm of H-151, 500 ng of LPS, and 5 μm of H-151 and 1 × 10⁸ particles/mL of EVs were added to the medium of the different treatment groups, respectively. to inflammation of the mechanism of action of EVs.

### Mycoplasma cytotoxicum test

200  μl of sample was collected into a PCR tube and centrifuged at 12000  rpm for 5 min, the supernatant was removed, 30  μl of pure water or PBS was added to resuspend the sample, the mixture was heated at 95 °C for 10 min, the mixture was cooled to 12 °C, and the mixture was finally centrifuged at 8,000  rpm for 2 min. The supernatant was used as the sample. The synthesized primers were diluted to a final concentration of 10 nmol/L to prepare the working solution for PCR reaction. A 1%–1.5% agarose gel was prepared in advance, the complete PCR reaction solution was added to the gel wells in sequence, and an appropriate marker for agarose gel electrophoresis was selected. After identification, the Mycoplasma detection result of sample 3D4/21 cell line was negative.

### Analyzing gene expression via qRT-PCR

Total RNA was extracted from mouse colon tissue using TRIzol reagent (Takara, Japan) and then reverse transcribed to complementary DNA (cDNA) using PrimeScript™ RT reagent Kit (Perfect Real Time) (RR037A) (Takara, Japan). Quantification of mRNA levels was performed using a standard quantitative reverse transcription polymerase chain reaction (RT-qPCR). RT-qPCR was performed on an Applied Biosystems 7500 Real-Time PCR System using TB Green® Premix Ex Taq™ (Tli RNaseH Plus), Bulk (RR420) (Takara, Japan). β-actin was used as an internal standard. The quantification of gene expression by RT-qPCR is dependent on the cycling threshold (CT) value, allowing relative assessment of mRNA levels. The expression levels were normalized to the β-actin expression levels and calculated by the 2-ΔΔCt method.

### Histopathological analysis

Colon tissue was fixed in 4% paraformaldehyde, paraffin-embedded and sectioned. The slides were dewaxed, rehydrated, and stained with hematoxylin and eosin. Blind measurements of intestinal crypt integrity, inflammatory cell infiltration, and mucosal injury were performed.

### Immunohistochemistry

The paraffin sections were placed in a 60 °C oven to bake the sections for 30–60 min, while they were sequentially placed in xylene I, II, and III for 10  min each, an ethanol gradient for 2  min each, and washed in water for 5  min. The sections were washed 3 times with PBS for 3min/time. The sections were infiltrated with preheated closed permeabilization solution for 30 min to reduce endogenous peroxidase activity. The cells were washed 3 times with PBS for 3 min/time. Formaldehyde fixation allows cross-linking between proteins and the closure of aldehyde groups, thereby losing antigenicity. Some non-specific binding sites were blocked with serum from the same source as the secondary antibody. Samples were incubated for 30 min at 37 °C in a warm box, and the excess serum was removed with filter paper. Then, 20 µl of diluted primary antibody was added to the tissue, which was incubated overnight at 4 °C or at 37 °C for 1–2 h. The samples were washed 3 times with PBS for 3 min/time. Diluted secondary antibody was added to the tissues, which were then incubated at 37 °C for 1–2 h and washed 3 times with PBS for 3 min/time. After color development with DAB-H_2_O_2_ for 10  min, the color solution is best used; at this time, observing whether the staining is obvious through a microscope, within 10  min can be terminated with distilled water color development. The samples were stained with Mayer’s hematoxylin for 30 s, washed with water, differentiated with hydrochloric acid–alcohol for 2 s, and immersed in running water for 15 min. The sections were dehydrated using a graded ethanol series (2 min at each concentration), rendered transparent by incubation in xylene for 5 min, and then sealed with neutral gum.

### Immunoblot analysis

A for adherent cells, the cells were rinsed with PBS for 2–3 times. After the last wash, pour out the PBS and try to dry the residual liquid; add an appropriate volume of RIPA lysis buffer (add various protease inhibitors within a few min before use) to the culture bottle for 3–5 min. During this period, shake the culture bottle repeatedly to make the reagent fully contact with the cells; scrape the cells with a cell scraper and transfer them to a 1.5 ml centrifuge tube; lyse on ice for 30 min, and use a pipette to blow repeatedly during this period to ensure that the cells are completely lysed; centrifuge at 12,000 rpm, 4 °C for 10 min, and collect the supernatant, which is the total protein solution.

For colon tissue, wash it 2–3 times with pre-cooled PBS to remove blood stains, cut it into small pieces and place it in a homogenization tube, add 2–4 mm homogenization beads, and add 10 times the volume of tissue RIPA lysis solution (add various protease inhibitors within a few min before use) for homogenization; take out the homogenization tube after homogenization, place the lysis solution on ice for 30 min, and shake it every 5 min to ensure that the tissue is completely lysed; centrifuge at 12,000 rpm, 4 °C for 10 min, collect the supernatant, which is the total protein solution. The undenatured protein solution was removed, the protein concentration was measured with the BCA protein concentration determination kit, and the protein solution was adjusted to the appropriate concentration. The protein solution was added to the reduced protein loading buffer, denatured in a boiling water bath for 15  min, and store it in a −20 °C refrigerator for later use.

Next, the gel was prepared for Western blot analysis according to the SDS‒PAGE gel preparation kit (Beyotime P0012A China), and the proteins were transferred to a polyvinylidene fluoride membrane activated with methanol (Beyotime P0965 China) using transfer buffer (0.025 M Tris, 0.192 M glycine, and 20% methanol) after electrophoresis. The polyvinylidene fluoride membrane was blocked in PBST solution containing 0.01% Tween 20 and 5% skim milk at room temperature for 1 h. For detection of STING (Cell Signaling 13647 America), TBK 1 (Cell Signaling 3504 America), and IRF 3 (Cell Signaling 4302 America), phosphorylated TBK 1 (Cell Signaling D52C2 America), and phosphorylated IRF 3 (Cell Signaling E6F7Q America), for normal proteins, the PVDF membrane was incubated overnight at 4 °C in TBST solution containing 1% BSA and primary antibody (Cell Signaling 9997S America). For phosphorylated proteins, the PVDF membrane was incubated overnight at 4 °C in a TBST solution containing 5% BSA and primary antibody. After incubation with the primary antibody, the PVDF membrane was then washed three times in TBST and then incubated for 1 h in TBST solution containing a goat anti-rabbit HRP-labeled secondary antibody (Thermo Fisher 31460 US) and 1% skim milk.

After washing, the PVDF membrane was removed and placed on absorbent paper. The liquid on the membrane was slightly dried, the membrane was placed on the shelf of the chemiluminescence instrument, and the membrane was exposed according to the instructions of BeyoECL SuperMoon (Beyotime P0018HS China). After exposure, the original photo was saved. The original image was analyzed with ImageJ.

### Liquid chromatography–mass spectrometry (LC–MS)

The samples were processed for small-molecule extraction by the addition of six volumes of chromatographic-grade methanol, followed by homogenization for 1 min and vortex mixing. After centrifugation at 10,000  rpm for 10  min at 4 °C, the supernatants were collected for LC–MS analysis. Chromatographic separation was performed on a Welch XB-C18 column (50 × 2.1 mm, 1.8 μm) using a binary mobile phase system consisting of solvent A (10% acetonitrile containing 0.1% formic acid) and solvent B (90% acetonitrile). The flow rate was set at 0.3 mL/min, and the column temperature was maintained at room temperature. The injection volume was 30  μL.

Mass spectrometric detection was carried out on a TSQ triple quadrupole mass spectrometer (Thermo Fisher Scientific) equipped with an electrospray ionization (ESI) source operating in both positive and negative ion switching modes. Data were acquired in multiple reaction monitoring (MRM) mode. The spray voltage was set to 5,500 V in positive mode and 4,500 V in negative mode, with an ion source temperature of 350 °C. Nitrogen was used as both the nebulizing gas and the collision gas. The total data acquisition time was 28.8 min per sample. An authentic c-di-AMP standard (Sigma–Aldrich) was dissolved in chromatographic-grade methanol to prepare a 1 mg/mL stock solution, which was subsequently diluted with chromatographic water to generate calibration standards ranging from 0 to 100 ng/mL. Quantification was performed using external standard calibration curves constructed with a 1/x weighting factor. Chromatographic peak integration and data processing were carried out using Xcalibur software (version 3.0; Thermo Fisher Scientific). The calibration curve exhibited excellent linearity over the tested concentration range (R² > 0.99).

### Co-inmunoprecipitation

To assess direct binding between STING and *S. hyointestinalis* EVs, we performed a cell-free pull-down assay using FLAG-tagged STING and biotin-labeled EVs. FLAG–STING was expressed in 3D4/21 cells and purified with anti-FLAG M2 affinity gel (Sigma-Aldrich 12352203 US). EVs were isolated by ultracentrifugation and surface-labeled with Biotin-NHS (Sigma–Aldrich 41116133 US) according to the manufacturer’s instructions, followed by removal of free biotin using desalting columns. For binding, purified FLAG–STING (2  µg) was incubated with biotin–EVs (20  µg total protein) in 500 µL binding buffer (20 mM Tris-HCl pH 7.4, 150 mM NaCl, 0.1% NP-40) for 2 h at 4 °C. Complexes were captured with anti-FLAG beads for 1 h at 4 °C, washed 4–5 times with binding buffer, and eluted in 2 × SDS sample buffer.

### Scanning electron microscope

In this study, to observe the morphological characteristics of *S. hyointestinalis*, scanning electron microscopy (SEM) was used for morphological identification. First, an appropriate amount of *S. hyointestinalis* bacterial fluid was removed after 24 h of incubation, and the organisms were collected by centrifugation (8,000  rpm, 5 min) and washed three times with 0.1 M PBS (pH 7.4) to remove the culture medium residue. The organisms were subsequently fixed with 2.5% glutaraldehyde and placed at 4°C for 12 h to maintain the cell structure. After fixation, the samples were again washed three times with PBS for 10 min each and then dehydrated sequentially with graded concentrations of 30%, 50%, 70%, 90%, and 100% ethanol for 10 min each step, and finally treated twice in 100% ethanol to ensure complete dehydration. The dehydrated samples were dried in a freeze dryer, and subsequently, the dried bacteriophage samples were adhered to a carrier stage and sprayed with a gold film using a metal sprayer to enhance electrical conductivity. Finally, the samples were observed under a scanning electron microscope (Hitachi, Japan), and the shape, arrangement, and surface characteristics of the bacterium were obtained from high-resolution images, and representative photographs were saved.

### Transmission electron microscope

The EVs samples obtained from the extraction were added dropwise onto the carbon film copper mesh, allowed to stand for 10 min at room temperature, then negatively stained with 2% phosphotungstic acid (pH 7.0) for 30 s, and then the excess staining solution was removed and naturally air-dried. Finally, the samples were placed under a transmission electron microscope to observe the vesicle morphology, and representative photographs were preserved.

### Laser confocal microscope

Macrophages at a concentration of 1 × 10^5^ cells ml^−1^ were cultured in 500  μl of medium and then incubated overnight (~18 h) at 37 °C and 5% CO_2_. For stimulation, 50  μl of exosomes (approximately 1011) were fluorescently labeled using the Exosome Tracking Kit (H-wayen China). The same washing procedure was performed for the PBS plus fluorescently labeled samples. Prior to stimulation, the macrophages were rinsed three times with PBS and then co-cultured for 3 h at 37 °C with 5% CO_2_ to enable them to phagocytose the exosomes. Finally, imaging and photography were performed using a Zeiss laser confocal microscope. The raw images were cropped by ZEISS ZEN 3.10 and assembled in Adobe Illustrator. Macrophage localization was quantified as nuclear (nuclear localization only), non-nuclear (EVs localization), or mixed (combination of nuclear and EVs).

### Immunophenotyping by flow cytometry and histopathology

Fresh tissue samples were taken, and the tissue was cut into small pieces and placed in the digest (collagenase type IV and 100 U DNase I in 100  mL Roswell Park Memorial Institute (RPMI) 1640 medium). The samples were either placed in a 37 °C water bath or homogenized using a tissue homogenizer for 1 h to ensure dissociation of the cells in the tissue. The samples were passed through a 70 mM filter, and the filtered cell precipitate was resuspended in buffer (PBS containing 1% BSA) to obtain a single-cell suspension. The cells were collected, counted and measured for viability, then resuspended and washed twice with 0.5% BSA/PBS solution, centrifuged at 400 g for 3 min to discard the supernatant; the cells were resuspended with FC receptor blocking solution, incubated for 1 h, then centrifuged and discarded the supernatant; the cells were resuspended with 0.5% BSA/PBS solution, homogenized into 96-well plates and then added with primary antibodies (PE/Cyanine5 anti-mouse CD45 Antibody, 103109; APC/Cyanine7 anti-mouse/human CD11b Antibody, 101225; FITC anti-mouse F4/80 Antibody, 123107; APC anti-mouse CD86 Antibody, 105011; Brilliant Violet 421™ anti-mouse CD206 (MMR) Antibody, 141717, all antibodies were obtained from Biolegend unless otherwise stated) dilution (0.5% BSA/PBS solution dilution), mixing well; 96-well plate to the oscillator, incubation at room temperature for 20 min; at the end of the incubation, centrifuged at 400 g for 5 min and discarded the supernatant, washed by resuspension with 0.5% BSA/PBS in each well, and washed by centrifugation at 400 g for 5 min and discarded the supernatant. After incubation, each well was washed with 0.5% BSA/PBS, resuspended at 400 × g, and centrifuged for 5 min, and the supernatant was discarded. The cells were washed with FACS buffer, and 7-aminoactinomycin D (Stem Cell Technologies, 75001.1) was added for live-cell staining. Flow cytometry was performed using BD FACSCelesta and analyzed using FlowJo 10 software (BD) (Figure S7a). Quantitative analysis was performed using FlowJo 10.9.0.

### Microscopy

In this study, *S. hyointestinalis* was morphologically characterized by scanning electron microscopy, *S. hyointestinalis* EVs were identified and characterized by projection electron microscopy, and the passage of EVs to macrophages was also assessed by laser confocal microscopy. The detailed steps and analytical methods are described in the Supplementary material.

### High-throughput 16S rRNA gene amplicon sequencing and analysis

Total microbial DNA was obtained from the intestinal contents via a genomic DNA extraction kit (AU46111-96, BioTeke, China) following the manufacturer's instructions. DNA was quantified by Qubit (Invitrogen, USA). PCR amplification was performed using V3–V4 primers 341F/805R (341F: 5′-CCTACGGGGNGGCWGCAG-3′; 805R: 5′-GACTACHVGGGGTATCTAATCC-3′). PCR amplification was performed under the following conditions: pre-denaturation at 98 °C for 30 s, denaturation at 98 °C for 10 s, annealing at 54 °C for 30 s, extension at 72 °C for 45 s, and 32 cycles. The last cycle was at 72 °C for 10 min. The PCR products were purified from AMPure XT Beads (Beckman Coulter Genomics, Danvers, MA, USA) and quantified by Qubit (Invitrogen, USA). Sequencing was performed using an Agilent 2100 Bioanalyzer (Agilent, USA) and the Illumina Library Quantification kit (Kapa Biosciences, Woburn, MA, USA), which were then further merged and analyzed on an Illumina NovaSeq instrument provided by Hangzhou LC-Bio Technology Ltd. in China. 6000 (PE250) was obtained from Hangzhou LC-Bio Technology Co.

Sequencing primers were removed from the de-multiplexed raw sequences using cutadapt (v1.9). Double-ended reads were then merged using FLASH (v1.2.8). Low-quality reads (quality scores < 20), short reads (<100 bp), and reads containing more than 5% “N” records were trimmed using the sliding window algorithm in fqtrim (v 0.94). Quality filtering according to fqtrim was performed to obtain high-quality clean labels. Chimeric sequences were filtered using Vsearch software (v2.3.4). Denoising and generation of amplicon sequence variants (ASVs) using DADA2. Species annotated sequence alignment was performed using the QIIME2 plug-in feature classifier against databases SILVA and NT-16S. The *α* and *β* diversities were calculated using QIIME2 and the relative abundance was used in bacterial taxonomy. LDA effect values were calculated using nsegata-lefse (LEfSe, LDA ≥ 3.0, *P* < 0.05). Other graphs were plotted using the R package (v3.4.4).

### Whole genome sequencing

The experimental workflow was performed according to the standard protocol provided by Oxford Nanopore Technologies (ONT). Whole-genome extraction and sequencing primarily included the following steps: 1. Extraction of high-quality genomic DNA, with purity, concentration, and integrity assessed using Nanodrop, Qubit, and 0.35% agarose gel electrophoresis. 2. Recovery of large DNA fragments using the BluePippin automated nucleic acid recovery system. 3. Library preparation (SQK-LSK109 ligation kit): DNA damage repair and end repair, magnetic bead purification, adapter ligation, magnetic bead purification; Qubit library quantification. 4. Sequencing run.

Information analysis primarily includes the following steps: 1. Raw data quality control, filtering low-quality and short reads. 2. Genome assembly involves performing de novo assembly on filtered reads and error correction on the assembled draft genome. 3. Genome component analysis, primarily including: repetitive sequences, coding genes, non-coding RNA, phage remnants, gene islands, CRISPR, etc. 4. Functional annotation, which primarily utilizes general databases such as Nr, UniProt, COG, and KEGG, and specialized databases such as CAZyme, PHI, and CARD. 5. Genome map analysis.

### Non-targeted metabolomics analysis

The collected samples were thawed on ice and metabolites were extracted with 80% methanol buffer. Liquid chromatography‒mass spectrometry (LC‒MS) analysis was subsequently performed. All samples were collected through the LC‒MS system according to the instrumental instructions. All chromatographic separations were performed using an UltiMate 3000 UPLC system (Thermo Fisher Scientific, Germany). An ACQUITY UPLC T3 column (Milford, USA) was used for the reversed-phase separation. The column temperature was maintained at 40 °C. The postphases were 5 mM ammonium acetate and 5 mM acetic acid and acetonitrile. The low flow rate was 0.3 mL/min, and the mobile phase consisted of methanol. The gradient elution conditions were as follows: 0–0.8 min, 2% acetonitrile; 0.8–2.8 min, 2%–70% acetonitrile; 2.8–5.6 min, 70%–90% acetonitrile; 5.6–6.4 min, 90%–100% acetonitrile; 6.4–8.0 min, 100% acetonitrile; 8.0–8.1 min, 100%–2% acetonitrile; and 8.1–10 min, 2% acetonitrile.

Metabolites eluted from the column were detected using a high-resolution tandem mass spectrometer, Q-Exactive (Thermo Scientific). Q-Exactive was operated in positive and negative ion modes. Parent ion spectra (m/z 70–1050) were acquired at 70,000 resolutions to achieve an AGC target value of 3e6. The maximum injection time was set to 100  ms. Data were acquired in DDA mode using a Top 3 configuration. The fragmentation spectrum was acquired at 17,500 resolutions to achieve an AGC target value of 1e5 with a maximum injection time of 80 ms. To assess the stability of the liquid chromatography‒mass spectrometry (LC‒MS) system throughout the acquisition process, one QC sample (a mixture of all samples) was collected after every 10 samples.

### Statistics

Data are presented as mean ± standard deviation (or median and interquartile range, where applicable). Statistical analyses were performed using GraphPad Prism 10.0 and SPSS 16.0 software. Comparisons between two groups were performed using Student's t-test. Comparisons among multiple groups were conducted using one-way/two-way ANOVA, followed by Tukey/Dunnett post-hoc tests when appropriate. For multiple repeated comparisons involving small sample sizes, *P*-values were adjusted using Bonferroni correction. For experiments with *n* = 3 groups, corrected *P* < 0.017 were considered statistically significant.

## Supplementary Material

Supplementary Material.docx Supplementary Material.docx

## Data Availability

The datasets presented in this study can be found in online repositories. The names and accession numbers of the repositories can be found in the article/Supplementary Materials. 16S rRNA amplicon sequencing data have been deposited at NCBI BioProjects (https://www.ncbi.nlm.nih.gov/bioproject/) under PRJNA1258382. Whole-genome sequencing data have been submitted to NCBI BioProjects (https://www.ncbi.nlm.nih.gov/bioproject/) under PRJNA1356376. Metabolomics data have been deposited at Metabolights (https://www.ebi.ac.uk/metabolights/) under MTBLS12450.
