# Supplementary material for: Microbial extracellular vesicles from min pigs remodel macrophage polarization via STING to sustain intestinal immune homeostasis
Source: Gut Microbes. 2026 Jan 27;18(1):2620126. doi: 10.1080/19490976.2026.2620126 (PMC12851393; doi:10.1080/19490976.2026.2620126)
Supplement: Supplementary Material.docx [file KGMI_A_2620126_SM4112.docx]

**Supporting Information to**

**Microbial Extracellular Vesicles from Min Pigs Remodel Macrophage Polarization via STING to Sustain Intestinal Immune Homeostasis**

Zhendong Sun^1^, Zichuan An^1^, Weichen Hong^1^, Chenpeng He^1^, Jiaxin Liu^1^, Yupu Wang^1^, Chenyu Xue^1, *^, Na Dong^1, *^

^1^ The Laboratory of Molecular Nutrition and Immunity, College of Animal Science and Technology, Northeast Agricultural University, Harbin, P. R. China

* Corresponding authors: cyxue@neau.edu.cn (Chenyu Xue), ndong@neau.edu.cn (Na Dong),

**Supplementary tables**

**Table. S1** Primer sequences for qRT-PCR of pig

| Primer name | Primer sequences |
| --- | --- |
| β-actin | F：TTCTAGGCGGACTTGCAGC |
|  | R：GCTTCTCAGCAGACAGGAGG |
| ZO-1 | F：GAAATACCTGACGGTGCTGC |
|  | R：GAGGATGGCGTTACCCACAG |
| Occludin | F：CAGGTGCACCCTCCAGATTG |
|  | R：TGGACTTTCAAGAGGCCTGG |
| TNF-α | F：GGCCCAAGGACTCAGATCAT |
|  | R：CTGTCCCTCGGCTTTGACAT |
| IL-12 | F：TCGTGCTGGAAGCTGTTCAC |
|  | R：GGAGGGTCTGGTTTGATGATGT |
| CD86 | F：TCGTTGTGTGTGGGATGGTG |
|  | R：TCATGGACTTCTGCTCTGTTCTT |
| Arg1 | F：TTTCTCCAAGGGTCAGCCAC |
|  | R：AGGGACATCAGCAAAGCACA |
| IL-10 | F：CGGCCCAGTGAAGAGTTTCT |
|  | R：GGCAACCCAGGTAACCCTTA |
| CD206 | F：GCTCCTGGAAGACAGCGAAT |
|  | R：AGTGGCAGGGGTTTCATCTG |

**Table. S2** Primer sequences for qRT-PCR of mice

| Primer name | Primer sequences |
| --- | --- |
| β-actin | F：TTCTAGGCGGACTTGCAGC |
|  | R：GCTTCTCAGCAGACAGGAGG |
| ZO-1 | F：GAAATACCTGACGGTGCTGC |
|  | R：GAGGATGGCGTTACCCACAG |
| Occludin | F：CAGGTGCACCCTCCAGATTG |
|  | R：TGGACTTTCAAGAGGCCTGG |
| IL-1β | F：GCCAGTCTTCATTGTTCAGGTTT |
|  | R：ATCTCTTTGGGGCCATCAGC |
| TNF-α | F：GGCCCAAGGACTCAGATCAT |
|  | R：CTGTCCCTCGGCTTTGACAT |
| iNOS | F：GAGCCCAGAGGGCTTTATCA |
|  | R：GGGAGTCTGGAGATTTCTTTGC |
| IL-12 | F：TCGTGCTGGAAGCTGTTCAC |
|  | R：GGAGGGTCTGGTTTGATGATGT |
| CD86 | F：TCGTTGTGTGTGGGATGGTG |
|  | R：TCATGGACTTCTGCTCTGTTCTT |
| IL-5 | F：GCTTCTGCATTTGAGTTTGCT |
|  | R：GAAATCATCAAGTTCCCATCGC |
| Arg1 | F：TTTCTCCAAGGGTCAGCCAC |
|  | R：AGGGACATCAGCAAAGCACA |
| IL-10 | F：CGGCCCAGTGAAGAGTTTCT |
|  | R：GGCAACCCAGGTAACCCTTA |
| CD206 | F：GCTCCTGGAAGACAGCGAAT |
|  | R：AGTGGCAGGGGTTTCATCTG |
| NF-κB | F: CTCTGGCACAGAAGTTGGGT |
|  | R: TCCCGGAGTTCATCTCATAGT |
| cGAS | F: GAGCAAAATGCTGCAGAAAAGA |
|  | R: CCTGGGTCCACATGTGAAAGA |

**Table. S3** Analysis of the difference in weight changes between Min pigs and DLY pigs during the 15-day nursery period

| Day | DN | DND | DCD | MN | MND | MCD |
| --- | --- | --- | --- | --- | --- | --- |
| Day1 | 25.88±25.82^a^ | 25.02±25.27^a^ | 25.24±25.03^a^ | 25.31±26.00^a^ | 25.53±25.43^a^ | 25.90±25.75^a^ |
| Day2 | 26.37±26.22^a^ | 25.44±25.69^a^ | 25.65±25.47^c^ | 25.73±26.48^ab^ | 26.03±25.85^a^ | 26.31±26.15^bc^ |
| Day3 | 26.85±26.64^a^ | 25.88±26.10^a^ | 26.08±25.96^b^ | 26.19±26.98^a^ | 26.47±26.33^a^ | 26.77±26.62^b^ |
| Day4 | 27.35±27.14^a^ | 26.33±26.53^a^ | 26.56±26.44^c^ | 26.59±27.47^b^ | 26.92±26.76^b^ | 27.18±27.02^c^ |
| Day5 | 27.80±27.64^a^ | 26.77±27.03^a^ | 27.03±26.89^c^ | 26.99±27.95^a^ | 27.36±27.16^a^ | 27.63±27.49^c^ |
| Day6 | 28.24±28.11^a^ | 27.25±27.48^a^ | 27.44±27.39^c^ | 27.48±28.38^a^ | 27.86±27.64^a^ | 28.11±27.92^c^ |
| Day7 | 28.66±28.57^a^ | 27.69±27.95^a^ | 27.88±27.79^d^ | 27.89±28.85^b^ | 28.36±28.04^b^ | 28.54±28.36^c^ |
| Day8 | 29.07±29.05^a^ | 28.15±28.45^a^ | 28.35±28.24^d^ | 28.33±29.30^b^ | 28.83±28.48^b^ | 29.00±28.83^c^ |
| Day9 | 29.49±29.49^a^ | 28.64±28.92^a^ | 28.79±28.71^d^ | 28.80±29.80^b^ | 29.24±28.90^b^ | 29.49±29.28^c^ |
| Day10 | 29.94±29.95^a^ | 29.12±29.32^b^ | 29.20±29.20^e^ | 29.22±30.26^bc^ | 29.68±29.37^c^ | 29.89±29.71^d^ |
| Day11 | 30.39±30.35^a^ | 29.57±29.79^c^ | 29.62±29.70^f^ | 29.72±30.70^b^ | 30.15±29.79^d^ | 30.3±30.13^e^ |
| Day12 | 30.80±30.82^a^ | 30.03±30.21^c^ | 30.04±30.10^e^ | 30.22±31.10^b^ | 30.57±30.22^c^ | 30.75±30.59^d^ |
| Day13 | 31.21±31.29^a^ | 30.46±30.65^c^ | 30.45±30.53^e^ | 30.64±31.52^b^ | 31.01±30.65^c^ | 31.24±31.05^d^ |
| Day14 | 31.62±31.76^a^ | 30.96±31.12^c^ | 30.92±31.01^e^ | 31.13±31.99^b^ | 31.43±31.09^c^ | 31.69±31.48^d^ |
| Day15 | 32.71 ± 0.36ᵃ | 31.34 ± 0.26ᵇ | 28.40 ± 0.32ᶜ | 28.26 ± 0.40ᶜ | 27.71 ± 0.22ᵈ | 25.67 ± 0.32ᵉ |

Note: a, b, c indicate significant differences in body surface temperature between different groups on the same day (*P* < 0.05) (n=12)

**Table. S4** Analysis of differences in feed intake between Min pigs and DLY pigs during the 15-day nursery period

| Day | DN (Kg) | DND (Kg) | DCD (Kg) | MN (Kg) | MND (Kg) | MCD (Kg) |
| --- | --- | --- | --- | --- | --- | --- |
| Day1 | 1.19±0.12ᵃᴬ | 1.22±0.08ᵃᴬ | 1.17±0.14ᵃᴬ | 1.21±0.10ᵃᴬ | 1.18±0.15ᵃᴬ | 1.20±0.09ᵃᴬ |
| Day2 | 1.25±0.07ᵃᴬ | 1.18±0.13ᵃᴬ | 1.21±0.06ᵃᴬ | 1.20±0.15ᵃᴬ | 1.26±0.09ᵃᴬ | 1.16±0.16ᵃᴬ |
| Day3 | 1.20±0.15ᵃᴬ | 1.27±0.09ᵃᴬ | 1.19±0.12ᵃᴬ | 1.28±0.08ᵃᴬ | 1.22±0.14ᵃᴬ | 1.25±0.07ᵃᴬ |
| Day4 | 1.27±0.09ᵃᴬ | 1.24±0.15ᵃᴬ | 1.26±0.10ᵃᴬ | 1.30±0.12ᵃᴬ | 1.31±0.08ᵃᴬ | 1.23±0.13ᵃᴬ |
| Day5 | 1.29±0.06ᵃᴬ | 1.30±0.10ᵃᴬ | 1.21±0.16ᵃᴬ | 1.33±0.07ᵃᴬ | 1.27±0.15ᵃᴬ | 1.28±0.09ᵃᴬ |
| Day6 | 1.31±0.14ᵃᴬ | 1.26±0.07ᵃᴬ | 1.30±0.11ᵃᴬ | 1.35±0.10ᵃᴬ | 1.34±0.06ᵃᴬ | 1.24±0.15ᵃᴬ |
| Day7 | 1.34±0.08ᵃᴬ | 1.32±0.12ᵃᴬ | 1.28±0.15ᵃᴬ | 1.33±0.13ᵃᴬ | 1.30±0.10ᵃᴬ | 1.29±0.11ᵃᴬ |
| Day8 | 1.36±0.13ᵃᴬ | 1.14±0.19ᵇᴮ | 1.03±0.26ᶜᴮ | 1.37±0.11ᵃᴬ | 1.19±0.22ᵇᴮ | 1.12±0.18ᵇᴮ |
| Day9 | 1.33±0.18ᵃᴬ | 1.20±0.14ᵇᴮ | 0.97±0.29ᶜᴮ | 1.41±0.09ᵃᴬ | 1.23±0.17ᵇᴮ | 1.09±0.24ᵇᴮ |
| Day10 | 1.42±0.10ᵃᴮ | 1.16±0.21ᵇᴮ | 1.11±0.20ᶜᴮ | 1.45±0.16ᵃᴮ | 1.21±0.26ᵇᴮ | 1.15±0.17ᵇᴮ |
| Day11 | 1.46±0.15ᵃᴮ | 1.23±0.13ᵇᴮ | 1.02±0.28ᶜᶜ | 1.43±0.12ᵃᴮ | 1.28±0.20ᵇᴮ | 1.13±0.23ᵇᴮ |
| Day12 | 1.48±0.11ᵃᴮ | 1.19±0.24ᵇᴮ | 1.16±0.22ᶜᶜ | 1.49±0.08ᵃᴮ | 1.32±0.16ᵇᴮ | 1.18±0.19ᵇᴮ |
| Day13 | 1.50±0.17ᵃᴮ | 1.28±0.18ᵇᴮ | 1.09±0.24ᶜᶜ | 1.55±0.13ᵃᴮ | 1.29±0.25ᵇᴮ | 1.21±0.15ᵇᴮ |
| Day14 | 1.53±0.09ᵃᶜ | 1.33±0.20ᵇᴮ | 1.14±0.19ᶜᶜ | 1.56±0.18ᵃᶜ | 1.40±0.14ᵇᴮ | 1.19±0.27ᵇᴮ |
| Day15 | 1.58±0.16ᵃᶜ | 1.31±0.17ᵇᴮ | 1.20±0.21ᶜᶜ | 1.63±0.11ᵃᶜ | 1.43±0.19ᵇᴮ | 1.27±0.20ᵇᴮ |

Note: a, b, c indicate significant differences in body surface temperature between different groups on the same day (*P* < 0.05) (n=12). A, B, C indicate significant differences in body surface temperature within the same group on different days (*P* < 0.05) (n=12).

**Table. S5** Analysis of body temperature differences between Min pigs and DLY pigs during the 15-day nursery period

| Day | DN (℃) | DND (℃) | DCD (℃) | MN (℃) | MND (℃) | MCD (℃) |
| --- | --- | --- | --- | --- | --- | --- |
| Day1 | 34.6±0.8ᵃᴬ | 34.5±0.9ᵃᴬ | 34.4±0.8ᵃᴬ | 34.8±0.7ᵃᴬ | 34.7±0.9ᵃᴬ | 34.6±0.8ᵃᴬ |
| Day2 | 34.7±0.9ᵃᴬ | 34.6±0.8ᵃᴬ | 34.5±0.9ᵃᴬ | 34.9±0.8ᵃᴬ | 34.8±0.9ᵃᴬ | 34.7±0.8ᵃᴬ |
| Day3 | 34.5±1.0ᵃᴬ | 34.6±0.9ᵃᴬ | 34.4±0.9ᵃᴬ | 34.8±0.8ᵃᴬ | 34.7±1.0ᵃᴬ | 34.6±0.9ᵃᴬ |
| Day4 | 34.8±0.8ᵃᴬ | 34.7±0.9ᵃᴬ | 34.6±0.8ᵃᴬ | 35.0±0.7ᵃᴬ | 34.8±0.9ᵃᴬ | 34.7±0.8ᵃᴬ |
| Day5 | 34.7±0.9ᵃᴬ | 34.8±0.8ᵃᴬ | 34.5±1.0ᵃᴬ | 34.9±0.8ᵃᴬ | 34.8±0.9ᵃᴬ | 34.6±0.9ᵃᴬ |
| Day6 | 34.9±0.8ᵃᴬ | 34.7±0.9ᵃᴬ | 34.6±0.9ᵃᴬ | 35.1±0.7ᵃᴬ | 34.9±0.8ᵃᴬ | 34.8±0.9ᵃᴬ |
| Day7 | 34.8±0.9ᵃᴬ | 34.6±1.0ᵃᴬ | 34.5±0.9ᵃᴬ | 35.0±0.8ᵃᴬ | 34.8±0.9ᵃᴬ | 34.7±0.9ᵃᴬ |
| Day8 | 34.7±0.9ᵃᴬ | 33.9±1.1ᵇᴮ | 32.8±1.4ᶜᴮ | 34.9±0.8ᵃᴬ | 34.1±1.2ᵇᴮ | 33.6±1.1ᵇᴮ |
| Day9 | 34.8±1.0ᵃᴬ | 34.0±1.2ᵇᴮ | 32.5±1.6ᶜᴮ | 35.0±0.9ᵃᴬ | 34.2±1.1ᵇᴮ | 33.7±1.3ᵇᴮ |
| Day10 | 34.9±0.9ᵃᴬ | 33.8±1.3ᵇᴮ | 32.9±1.5ᶜᴮ | 35.1±0.8ᵃᴬ | 34.0±1.4ᵇᴮ | 33.8±1.2ᵇᴮ |
| Day11 | 35.0±0.8ᵃᴬ | 34.1±1.1ᵇᴮ | 32.6±1.7ᶜᶜ | 35.2±0.7ᵃᴬ | 34.3±1.3ᵇᴮ | 33.9±1.4ᵇᴮ |
| Day12 | 35.1±0.9ᵃᴬ | 34.0±1.4ᵇᴮ | 33.0±1.5ᶜᶜ | 35.3±0.8ᵃᴬ | 34.4±1.2ᵇᴮ | 34.1±1.3ᵇᴮ |
| Day13 | 35.0±1.0ᵃᴬ | 34.2±1.2ᵇᴮ | 32.7±1.6ᶜᶜ | 35.4±0.9ᵃᴬ | 34.3±1.4ᵇᴮ | 34.0±1.5ᵇᴮ |
| Day14 | 35.2±0.9ᵃᴬ | 34.3±1.3ᵇᴮ | 32.9±1.4ᶜᶜ | 35.5±0.8ᵃᴬ | 34.6±1.2ᵇᴮ | 34.2±1.4ᵇᴮ |
| Day15 | 35.3±1.0ᵃᴬ | 34.4±1.2ᵇᴮ | 33.1±1.5ᶜᶜ | 35.6±0.9ᵃᴬ | 34.7±1.3ᵇᴮ | 34.3±1.6ᵇᴮ |

Note: a, b, c indicate significant differences in body surface temperature between different groups on the same day (*P* < 0.05) (n=12). A, B, C indicate significant differences in body surface temperature within the same group on different days (*P* < 0.05) (n=12).

**Table. S6** Analysis of body weight changes of mice in each group during 15 days of feeding

| Day | Con | Cold | *S. hyointestinalis* | Cold +  *S. hyointestinalis* | Cold + DSS | Cold + DSS +  *S. hyointestinalis* |
| --- | --- | --- | --- | --- | --- | --- |
| Day1 | 19.79 ± 0.53^a^ | 19.94 ± 0.57^a^ | 19.84 ± 0.63^a^ | 20.06 ± 0.66^a^ | 19.86 ± 0.70^a^ | 19.69 ± 0.54^a^ |
| Day2 | 20.06 ± 0.48^a^ | 20.28 ± 0.61^a^ | 20.26 ± 0.65^a^ | 20.44 ± 0.69^a^ | 20.13 ± 0.72^a^ | 20.05 ± 0.59^a^ |
| Day3 | 20.34 ± 0.55^a^ | 20.63 ± 0.57^a^ | 20.66 ± 0.68^a^ | 20.81 ± 0.74^a^ | 20.42 ± 0.78^a^ | 20.49 ± 0.63^a^ |
| Day4 | 20.66 ± 0.53^a^ | 20.94 ± 0.54^a^ | 21.07 ± 0.75^a^ | 21.17 ± 0.75^a^ | 20.74 ± 0.73^a^ | 20.85 ± 0.60^a^ |
| Day5 | 20.89 ± 0.51^c^ | 21.26 ± 0.5^abc^ | 21.51 ± 0.7^ab^ | 21.6 ± 0.74^a^ | 21.01 ± 0.71^bc^ | 21.29 ± 0.56^abc^ |
| Day6 | 21.17 ± 0.62^b^ | 21.56 ± 0.52^ab^ | 21.95 ± 0.7^a^ | 21.96 ± 0.77^a^ | 21.27 ± 0.72^b^ | 21.74 ± 0.54^ab^ |
| Day7 | 21.47 ± 0.6^b^ | 21.90 ± 0.58^ab^ | 22.33 ± 0.75^a^ | 22.35 ± 0.78^a^ | 21.57 ± 0.71^b^ | 22.18 ± 0.63^a^ |
| Day8 | 21.81 ± 0.61^bc^ | 21.59 ± 0.53^bc^ | 22.72 ± 0.75^a^ | 22.11 ± 0.8^b^ | 21.24 ± 0.76^c^ | 21.93 ± 0.63^b^ |
| Day9 | 22.09 ± 0.62^b^ | 21.27 ± 0.55^cd^ | 23.09 ± 0.73^a^ | 21.85 ± 0.8^bc^ | 20.92 ± 0.73^d^ | 21.67 ± 0.62^bc^ |
| Day10 | 22.42 ± 0.62^b^ | 20.91 ± 0.58^d^ | 23.48 ± 0.7^a^ | 21.59 ± 0.8^c^ | 20.55 ± 0.77^d^ | 21.41 ± 0.63^cd^ |
| Day11 | 22.7 ± 0.63^b^ | 20.52 ± 0.59^d^ | 23.88 ± 0.68^a^ | 21.36 ± 0.8^c^ | 20.24 ± 0.76^d^ | 21.16 ± 0.62^c^ |
| Day12 | 22.93 ± 0.69^b^ | 20.16 ± 0.55^d^ | 24.29 ± 0.72^a^ | 21.12 ± 0.82^c^ | 19.92 ± 0.79^d^ | 20.92 ± 0.63^c^ |
| Day13 | 23.21 ± 0.67^b^ | 19.75 ± 0.57^d^ | 24.69 ± 0.74^a^ | 20.87 ± 0.83^c^ | 19.34 ± 0.8^d^ | 20.52 ± 0.64^c^ |
| Day14 | 23.57 ± 0.69^b^ | 19.36 ± 0.57^d^ | 25.07 ± 0.8^a^ | 20.63 ± 0.82^c^ | 18.54 ± 0.79^e^ | 19.91 ± 0.64^d^ |
| Day15 | 23.86 ± 0.71^b^ | 19.01 ± 0.59^d^ | 25.4 ± 0.78^a^ | 20.38 ± 0.84^c^ | 17.54 ± 0.82^e^ | 19.11 ± 0.64^d^ |

**Table. S7** LC-MS Determination of Cyclic Dinucleotide Analogs in Exosomes from *S. hyointestinalis*

| Group | c-di=AMP(ng/mL) |
| --- | --- |
| *S. hyointestinalis* EVs | 71.28 |
| Mock EVs | ns |
| EVs free supernatant | 5.33 |
| *S. hyointestinalis EVs+PDE* | 17.36 |

**Table. S8** 3D4/21 Cell Line Species Identification Amplification Curve

| Inspection Lane | Positive Control | Negative Control |
| --- | --- | --- |
| Pig and Cattle Inspection Lane  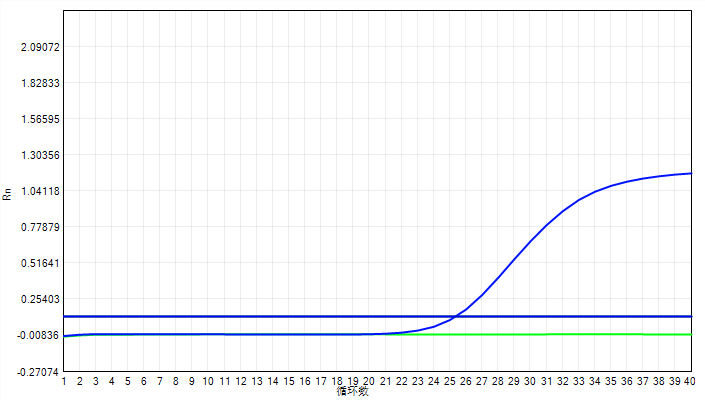 | Positive Control A  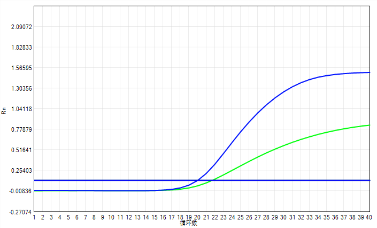 | Negative Control A  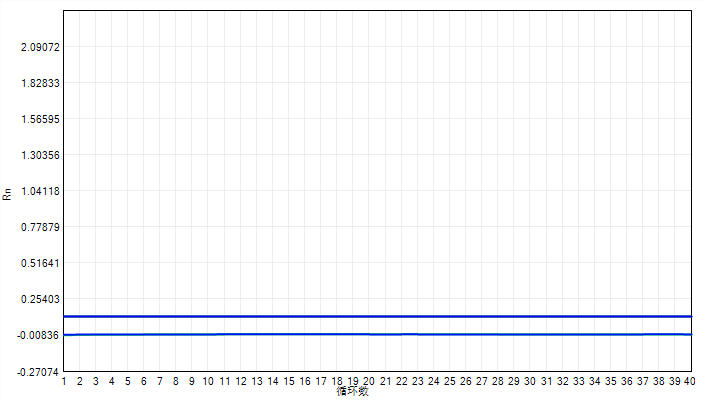 |
| Dog and Rabbit Inspection Lane  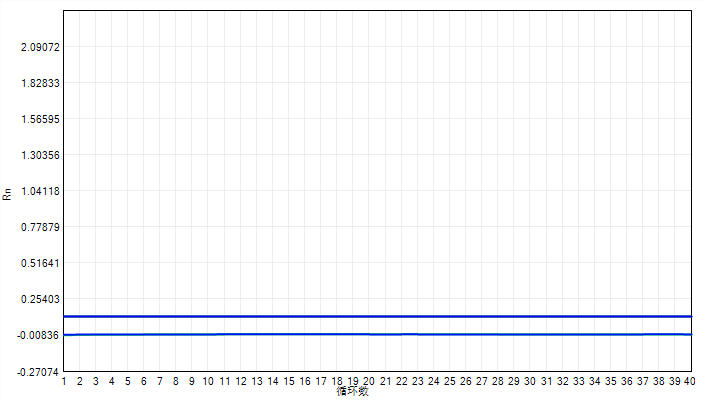 | Positive Control B  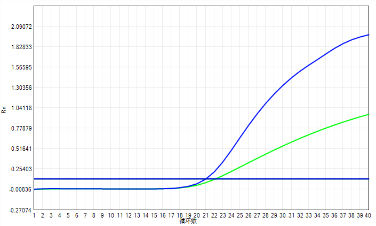 | Negative Control B  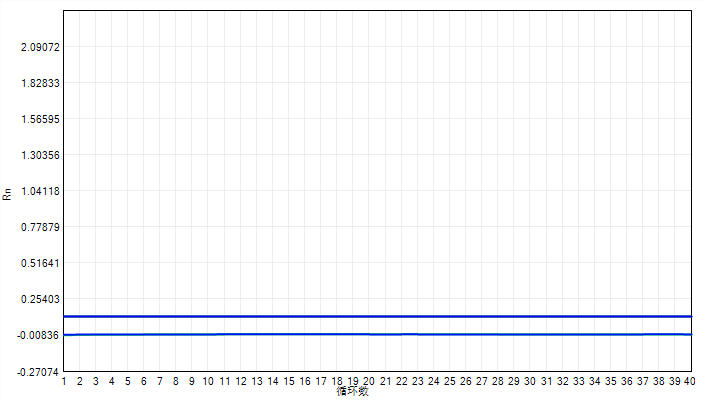 |
| Cat and Wart Detection Channel  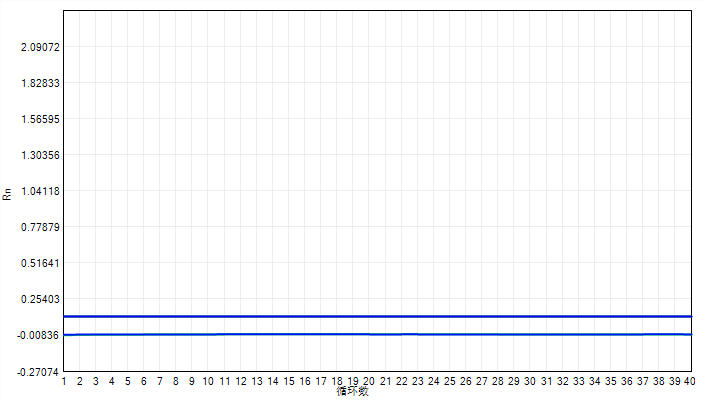 | Positive Control B  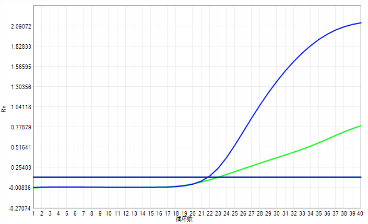 | Negative Control B  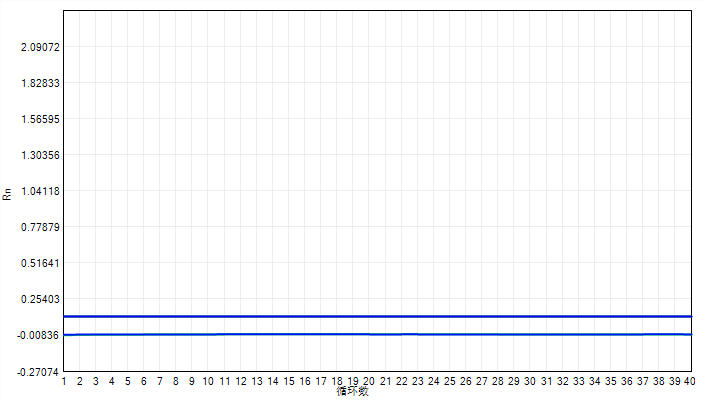 |

Note: Pig and Cow Detection Channel 1: Cow (HEX Green), Pig (FAM Blue); Positive Control A: Cow (HEX Green), Pig (FAM Blue); Negative Control A: Cow (HEX Green), Pig (FAM Blue). Dog and Rabbit Detection Channel 2: Dog (HEX Green), Rabbit (FAM Blue); Positive Control B: Dog (HEX Green), Rabbit (FAM Blue); Negative Control B: Dog (HEX Green), Rabbit (FAM Blue). Cat and Monkey Detection Channel 3: Cat (HEX Green), Monkey (FAM Blue); Positive Control C: Cat (HEX Green), Monkey (FAM Blue); Negative Control C: Cat (HEX Green), Monkey (FAM Blue).

**Table. S9** Summary of Ct Values from QPCR Detection Results

|  | Pig | Cow | Dog | Rabbit | Monkey | Cat |
| --- | --- | --- | --- | --- | --- | --- |
| 3D4/21 | 25.33 | undet | undet | undet | undet | undet |
| Positive Control | 20.01 | 21.68 | 22.16 | 21.04 | 21.77 | 22.99 |
| Negative Control | undet | undet | undet | undet | undet | undet |

**Supplementary figures**


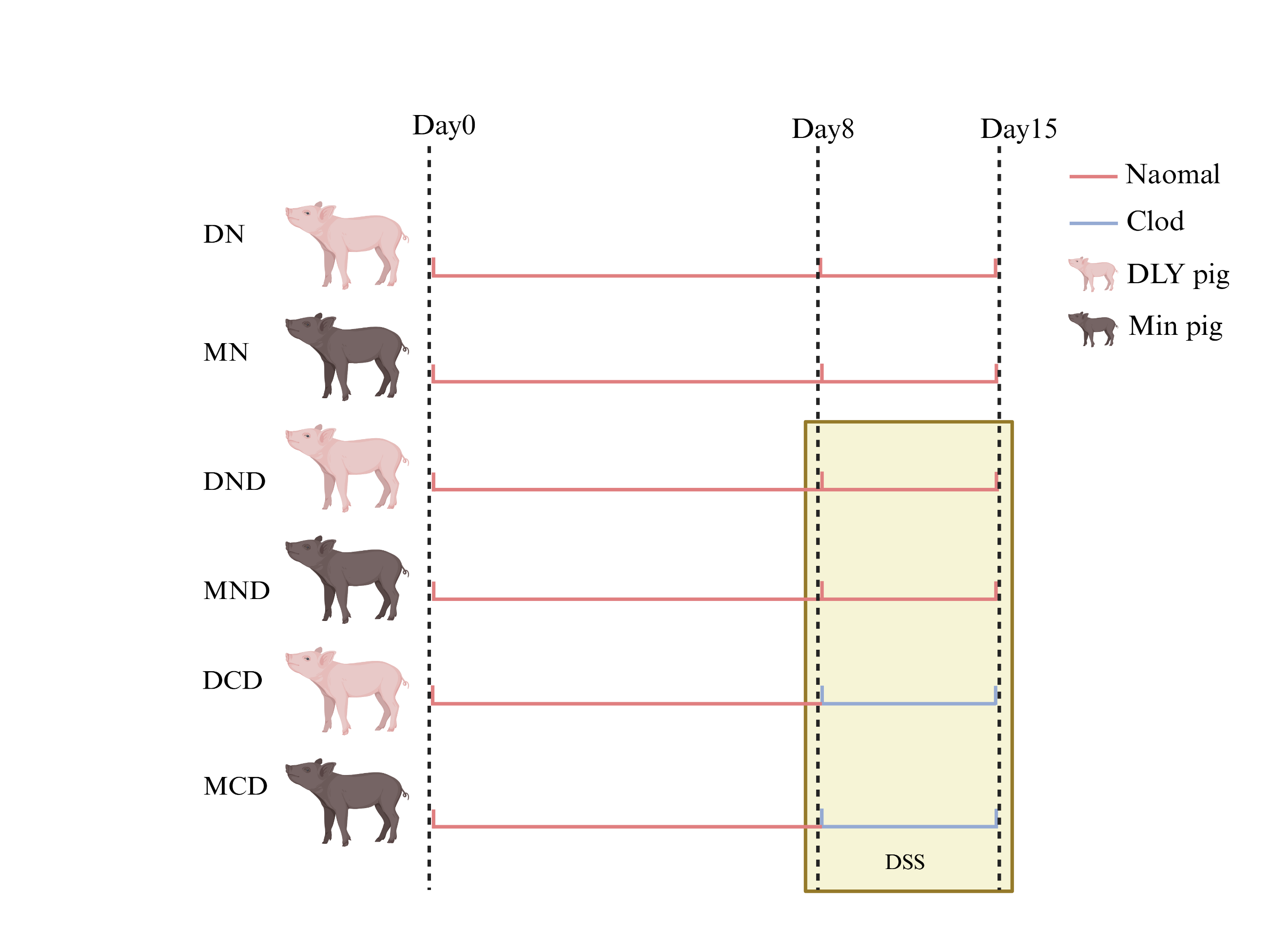


**Figure. S1** **Pig feeding program.** Two pig breeds (Min pigs and DLY pigs) were divided into three groups: a normothermic group (housed at room temperature for 15 days), a normothermic‐stimulated group (housed at room temperature for 15 days with 3% DSS in the drinking water from day 8), and a cold‐stress‐stimulated group (housed at 4–8℃ for 15 days with 3% DSS in the drinking water from day 8). Each group comprised six biological replicates (n = 6).


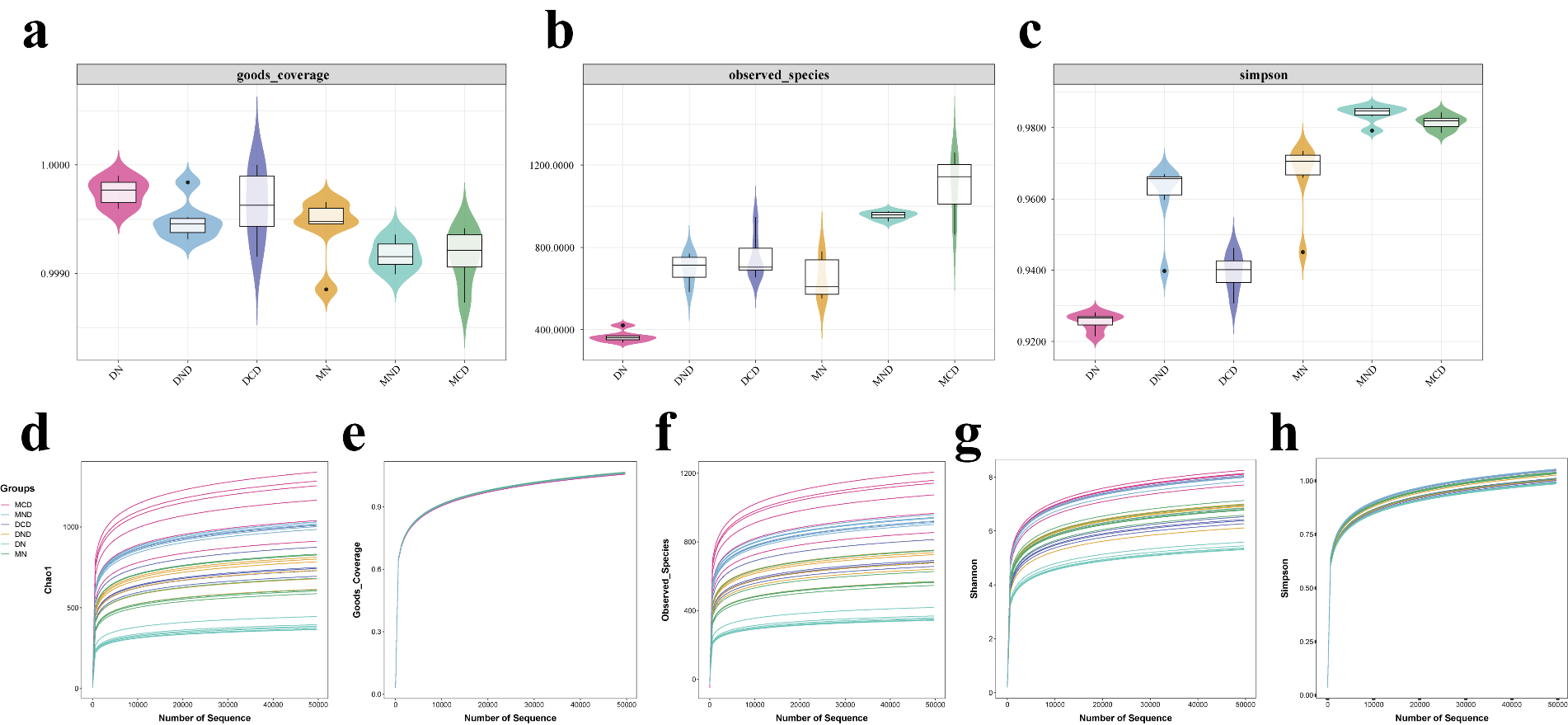


**Figure. S2 Analysis of alpha diversity in the intestinal flora of pigs.** Alpha diversity scores (n=6) for **(a)** Goods_coverage index, **(b)** Observe_species index, and **(c)** simpson index; analysis of sparsity between alpha diversity **(d-h)** sequence numbers and OTU numbers of the intestinal flora of Min pigs and DLY pigs.


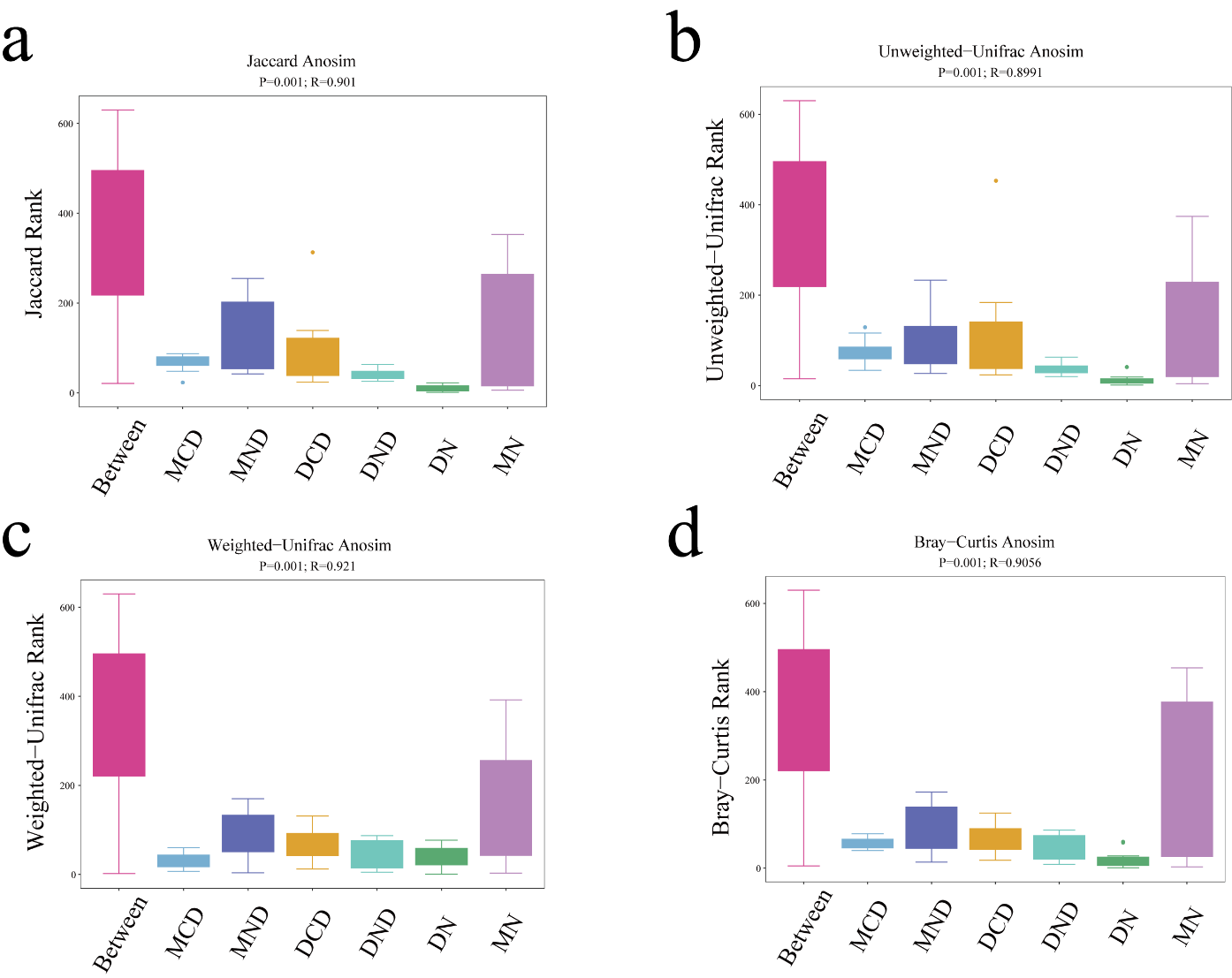


**Figure. S3 Anosim analysis of beta diversity in porcine intestinal flora.** Beta distance matrix analysis of **(a)** Jaccard, **(b)** Unweighted, **(c)** Weighted_unifrac and **(d)** Bray_curtis.


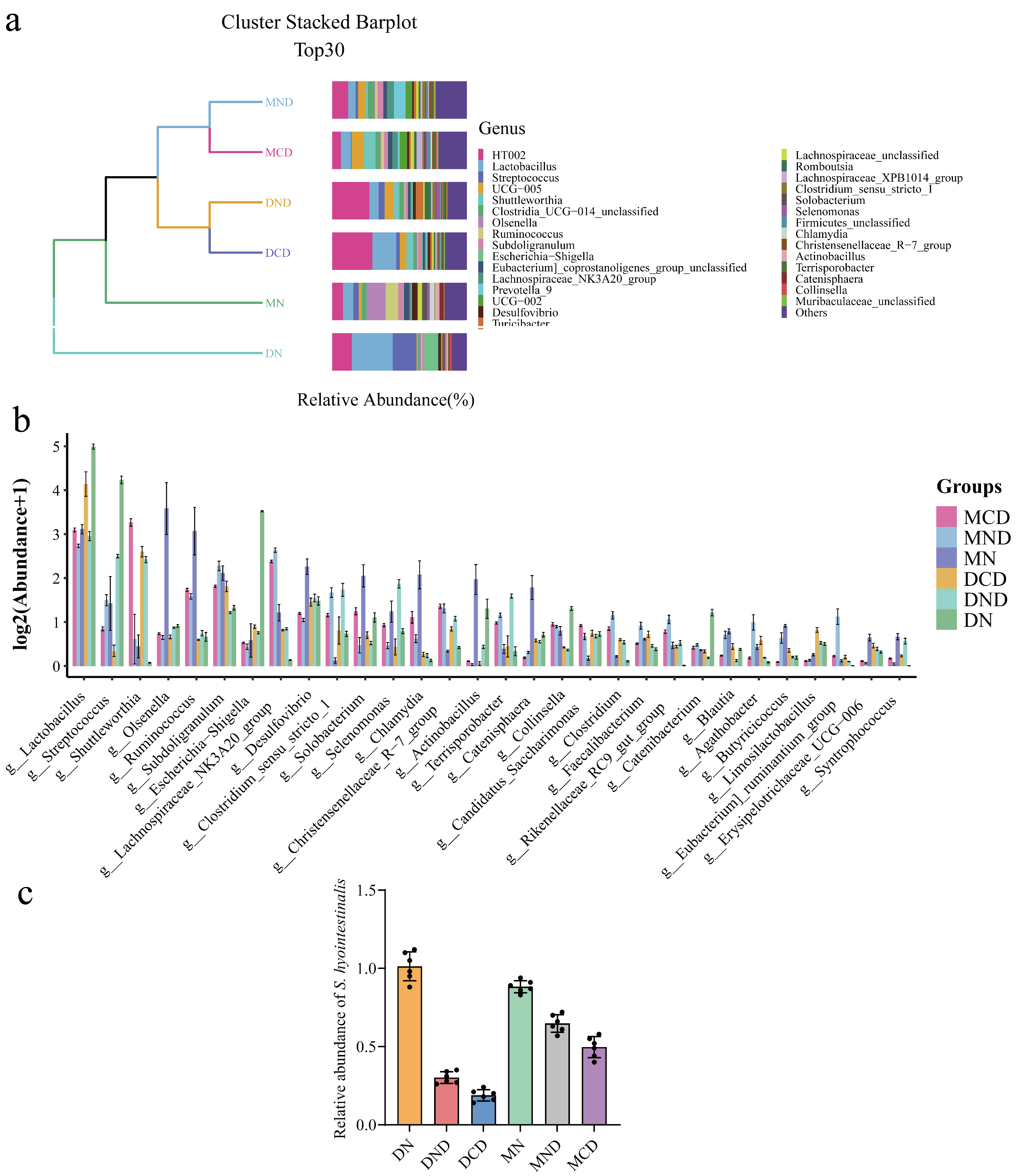


**Figure. S4 Differences in gut microbial composition between the two breeds of pigs under different conditions were determined by 16S rRNA gene sequencing.** Clustered stacked bar graphs show differences and associations in microbial composition at genus level **(a)** in individuals or groups; bar graphs show differences in microbial abundance at genus level **(b)** in individuals or groups. **(c)** Relative abundance of *S. hyointestinalis* in pig cecal contents.


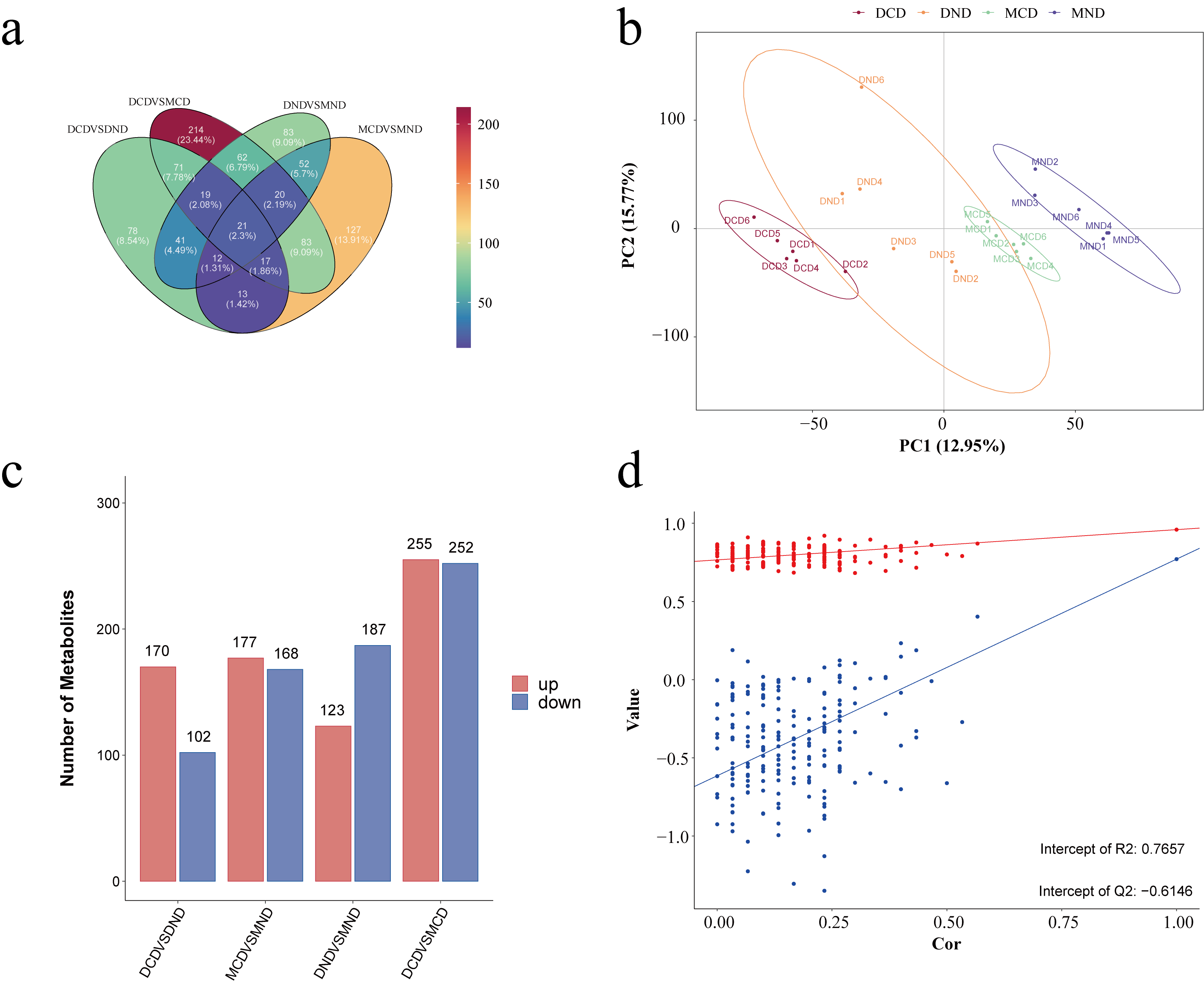


**Figure. S5 Differences in intestinal metabolites between two breeds of pigs under different conditions.** **(a)** Multi-comparison group Wayne plots showing the proportion of identical versus differential metabolites in different experimental groups; **(b)** PLSDA score plots of different treatment groups showing the degree of dispersion of metabolite differences between the groups; **(c)** Multi-comparison group statistical bar charts counting the number of metabolites that were up-regulated and down-regulated in the groups; and **(d)** PLSDA model-testing plots checking the reliability of the PLSDA analyses.


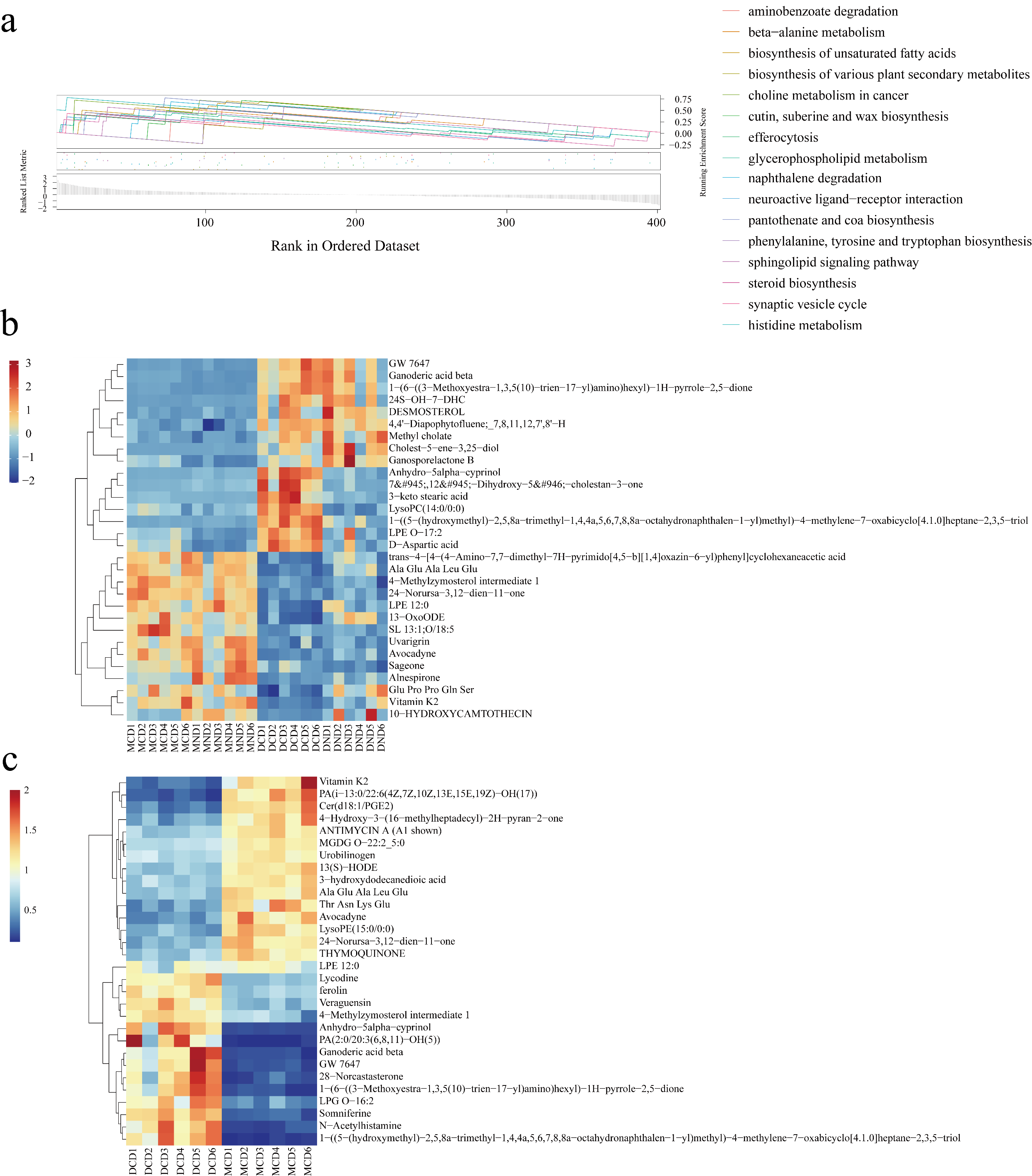


**Figure. S6 Functional analysis of intestinal differential metabolites in each experimental group. (a)** GSEA enrichment analysis showing differences in functions such as synaptic vesicle cycling, cytocytosis and steroids. **(b)** Heatmap of differential functional metabolite analysis in each group. **(c)** Differential Metabolite Analysis of DCD vs. MCD.


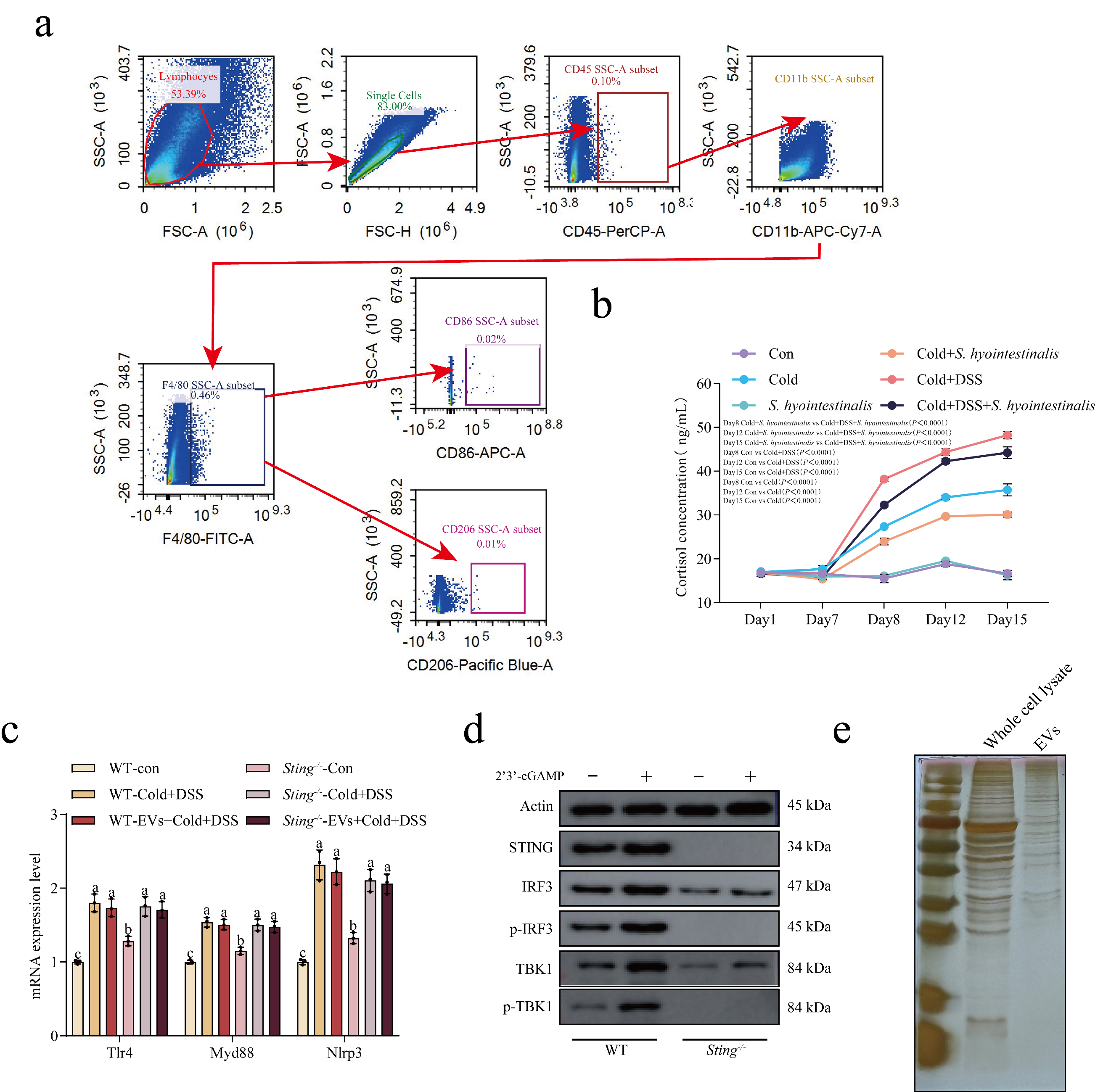


**Figure. S7 (a)**Gating strategy for flow cytometry analysis of mouse colon tissue. **(b)** ELISA assay for cortisol levels in mouse serum. **(c)** Relative expression levels of cytokines and inflammasomes in mouse colon tissue. **(d)** Expression levels of STING signaling pathway proteins in mouse colon tissue. **(e)** SDS-PAGE with silver staining for protein detection in EVs.


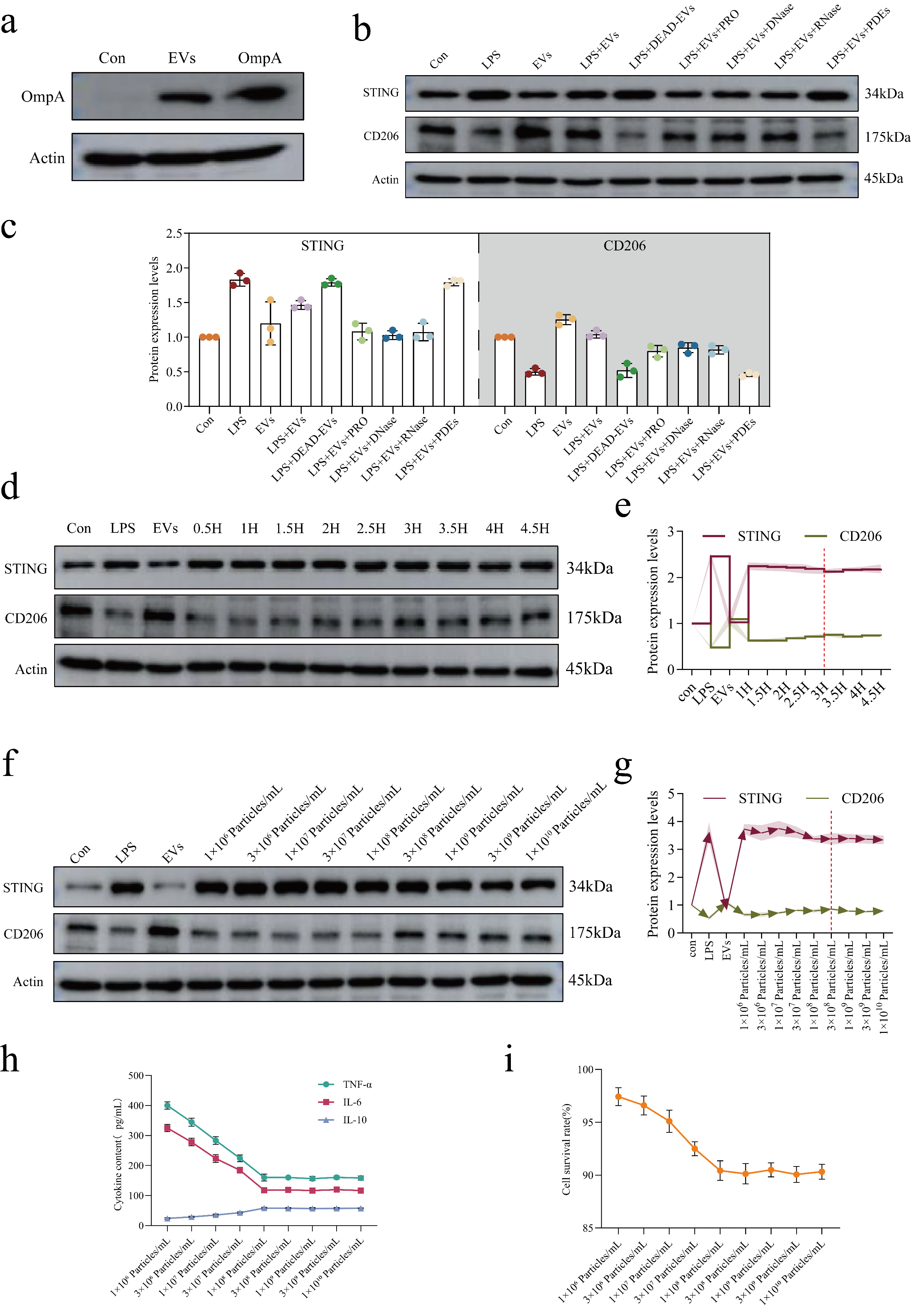


**Fig. S8 Verification of EV Activity and Pollution (a)** Identification of the surface marker OmpA on EVs via Western blot. **(b, c)** Western blot analysis of STING expression and polarization in LPS-induced 3D4/21 cells following treatment with EVs subjected to heat inactivation, protease, DNase, RNase, or phosphodiesterase. Statistical analysis of grayscale values. **(d, e)** Immunoblotting analysis of STING expression and polarization in LPS-induced 3D4/21 cells co-cultured with EVs for 4.5 hours, and in LPS-induced 3D4/21 cells co-cultured with EVs for 0.5h, 1h, 1.5h, 2h, 2.5h, 3h, 3.5h, 4h, and 4.5h. and statistical analysis of gray values. **(f, g)** Immunoblotting analysis of STING expression and polarization in 3D4/21 cells co-cultured with EVs for 3 h, and in LPS-induced 3D4/21 cells treated with EVs at concentrations of 1×10⁶, 3×10⁶, 1×10⁷, 3×10⁷, 1×10⁸, 3×10⁸, 1×10⁹, 3×10⁹ and 1×10¹⁰ particles/mL, and LPS-induced 3D4/21 cells. Statistical analysis of grayscale values. (h) Measurement of functional cytokine (TNF-α, IL-6, and IL-10) levels in RAW cells. (i) RAW cell survival rate.


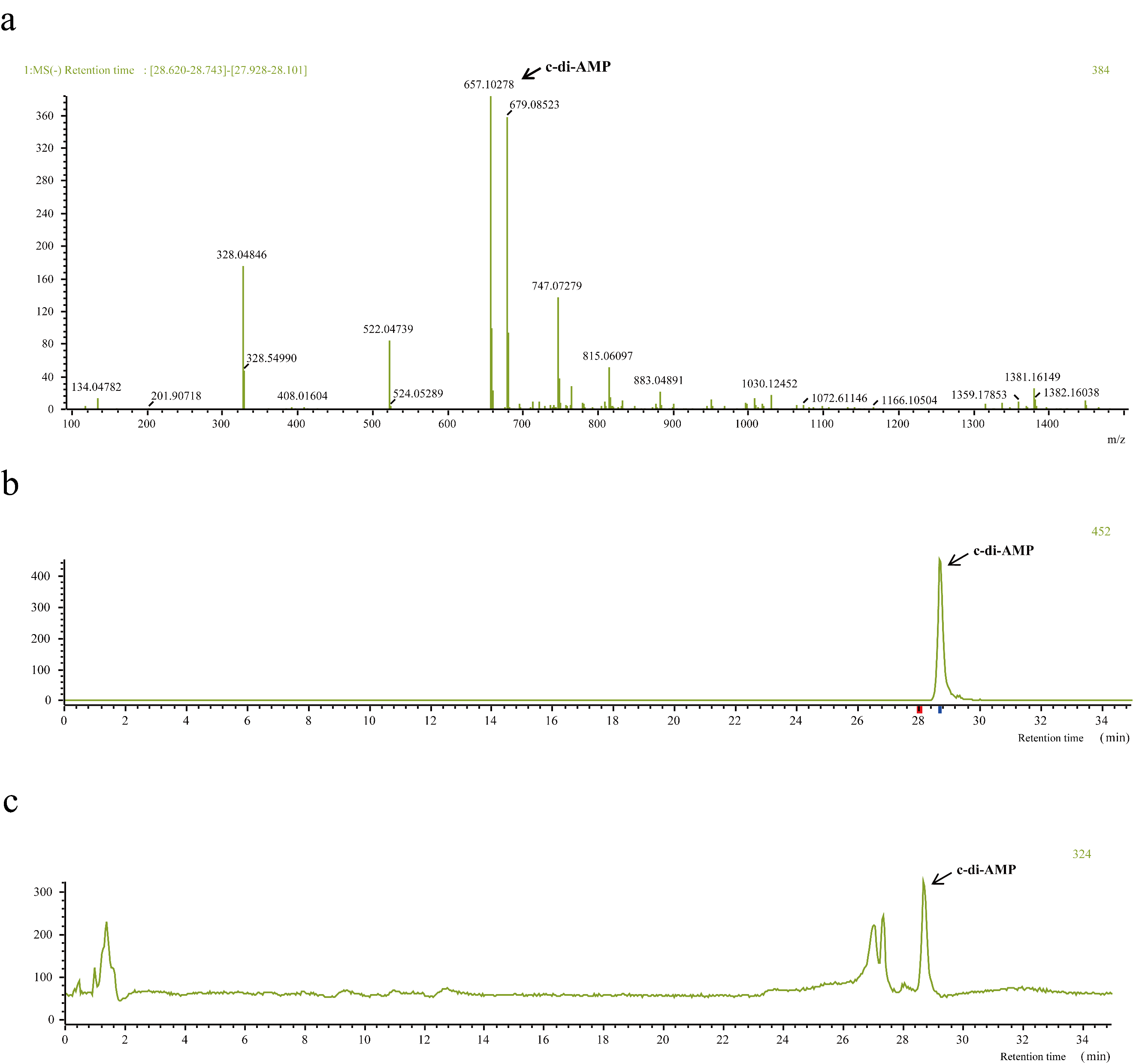


**Fig. S9 LC–MS Determination of Cyclic Dinucleotide Analogs in EVs (a)** Mass spectrum of c-di-AMP in EVs samples. **(b)** Ion flow diagram of c-di-AMP standard. **(c)** Ion flow diagram of EVs.


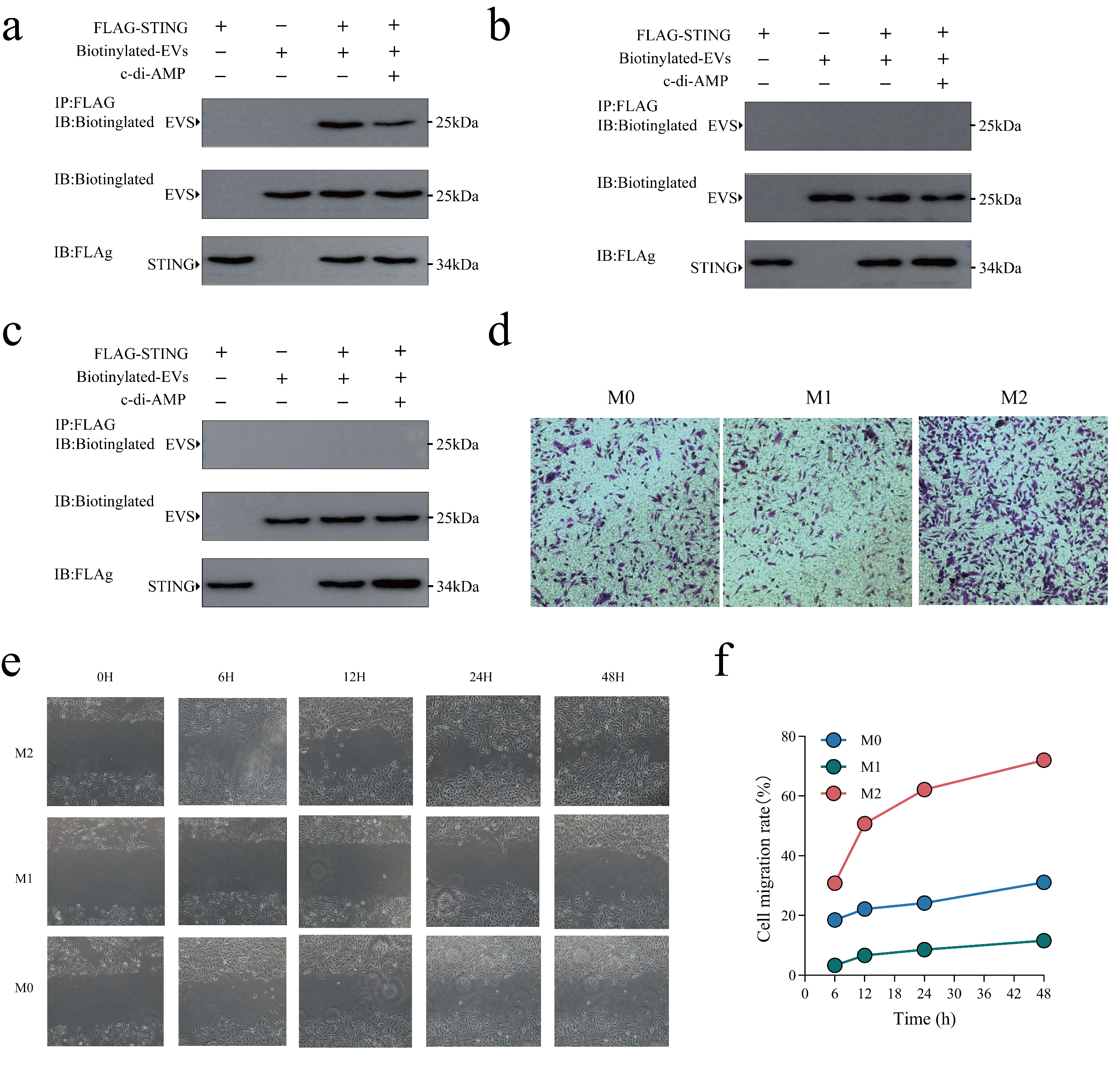


**Fig. S10 Regulatory Mechanisms of EVs on the STING Pathway and the Effect of Macrophage Polarization on Intestinal Epithelial Cell Migration (a)** Immunoprecipitation validated that *S. hyointestinalis* EVs can bind to STING protein. **(b)** Immunoprecipitation validated that *E. coli* EVs can bind to STING protein. **(c)** Immunoprecipitation validated that *Lactobacillus* EVs can bind to STING protein. **(d)** Photograph of non-contact Transwell co-culture between IPEC-J2 cells and 3D4/21 cells (3D4/21 cells stained with H&E). **(e)** Non-contact Transwell co-culture (0.4 μm pore size) simulates paracrine regulation of IPEC-J2 cells (upper chamber) by 3D4/21 cells (basal/intrinsic layer side) via secreted factors. Observation of IPEC-J2 cell scratch repair rate. **(f)** Statistical analysis of IPEC-J2 cell scratch repair rate.


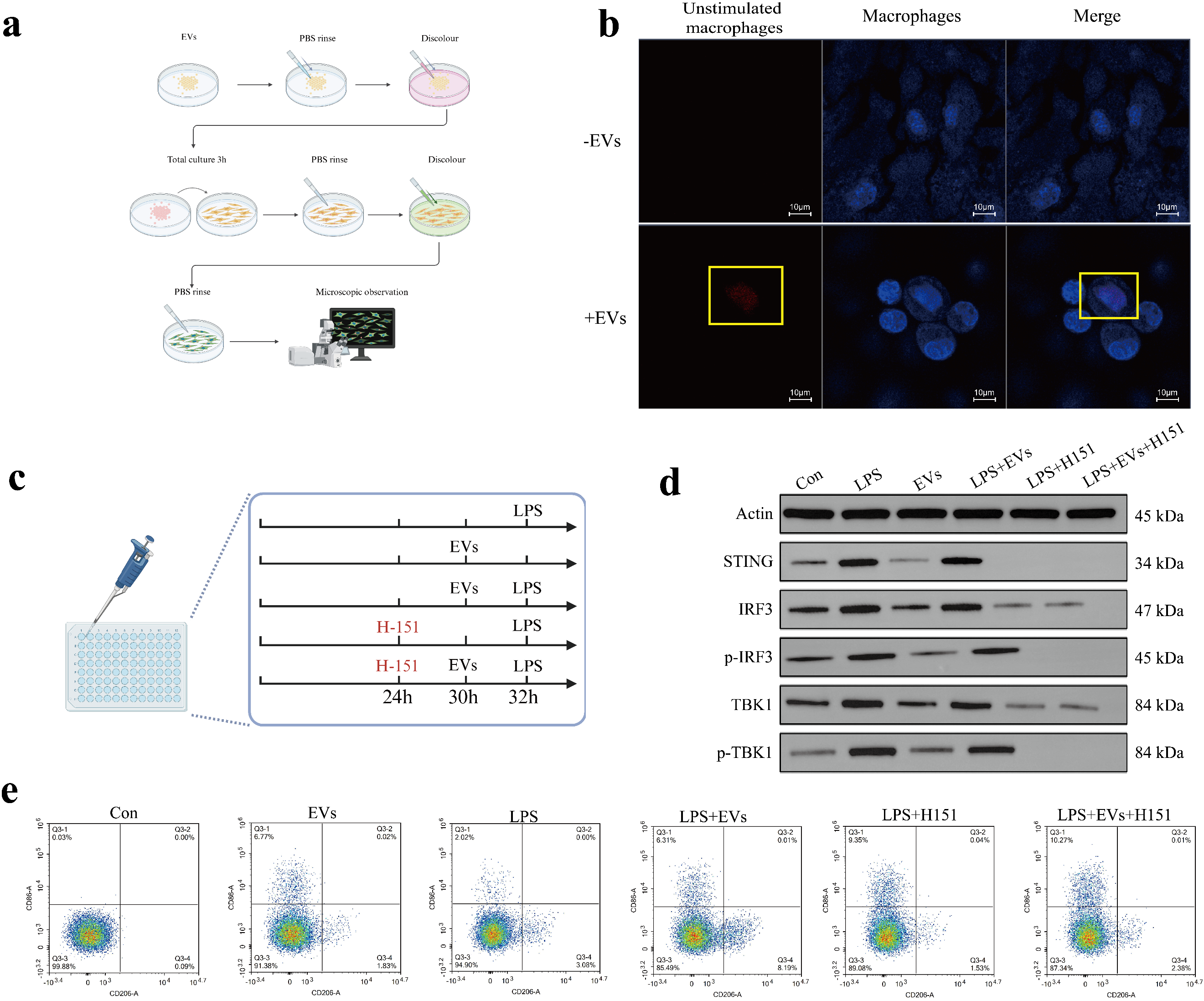


**Fig. S11 Invasion of EVs into macrophages regulates the STING pathway to influence their polarization (a)** Experimental design for investigating whether EVs can enter macrophages. **(b)** Representative laser confocal microscopy images of macrophages stimulated with red fluorescently labeled EVs and with unstimulated macrophages; macrophages were stimulated for 3 h prior to imaging. **(c)** Experimental design for investigating the mechanism of target-regulated action of EVs. **(d)** STING pathway-induced immunoblotting of macrophages with PBS, LPS, EVs and H151 (n=3). **(e)** PBS, LPS, EVs, and H151-induced polarization flow cytometry of macrophages (n=3).


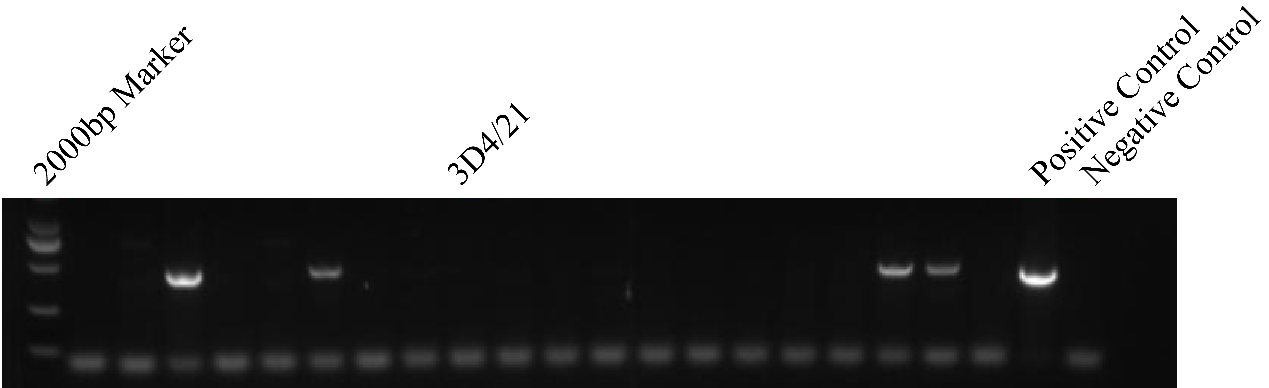


**Fig. S12 3D4/21 Cell Mycoplasma Detection Results**
